# A Review on the Potential Use of Medicinal Plants From Asteraceae and Lamiaceae Plant Family in Cardiovascular Diseases

**DOI:** 10.3389/fphar.2020.00852

**Published:** 2020-06-05

**Authors:** Jennifer Michel, Nur Zahirah Abd Rani, Khairana Husain

**Affiliations:** Drug and Herbal Research Centre, Faculty of Pharmacy, Universiti Kebangsaan Malaysia, Kuala Lumpur, Malaysia

**Keywords:** cardiovascular diseases, coronary heart disease, stroke, Asteraceae, Lamiaceae, hypertension, heart failure, medicinal plants

## Abstract

Cardiovascular diseases are one of the most prevalent diseases worldwide, and its rate of mortality is rising annually. In accordance with the current condition, studies on medicinal plants upon their activity on cardiovascular diseases are often being encouraged to be used in cardiovascular disease management, due to the availability of medicinal values in certain dedicated plants. This review was conducted based on two plant families, which are Asteraceae and Lamiaceae, to study on their action in cardiovascular disease relieving activities, to review the relationship between the phytochemistry of Asteraceae and Lamiaceae families and their effect on cardiovascular diseases, and to study their toxicology. The medicinal plants from these plant family groups are collected based on their effects on the mechanisms that affect the cardiovascular-related disease which are an antioxidant activity, anti-hyperlipidemic or hypocholesterolemia, vasorelaxant effect, antithrombotic action, and diuresis effect. In reference to various studies, the journals that conducted *in vivo* or *in vitro* experiments, which were used to prove the specific mechanisms, are included in this review. This is to ensure that the scientific value and the phytochemicals of the involved plants can be seen based on their activity. As a result, various plant species from both Asteraceae and Lamiaceae plant family have been identified and collected based on their study that has proven their effectiveness and uses in cardiovascular diseases. Most of the plants have an antioxidant effect, followed by anti-hyperlipidemia, vasorelaxant, antithrombotic, and diuretic effect from the most available to least available studies, respectively. These are the mechanisms that contribute to various cardiovascular diseases, such as heart attack, stroke, coronary heart disease, and hypertension. Further studies can be conducted on these plant species by identifying their ability and capability to be developed into a new drug or to be used as a medicinal plant in treating various cardiovascular diseases.

## Introduction

Cardiovascular diseases (CVD) are known as the most frequent and common cause of death worldwide. World Health Organization (WHO) reported that the total number of people who died from CVDs in 2012 was estimated to be around 17.5 million people representing 31% worldwide population. Disorders of heart and blood vessels, including coronary heart disease (CHD), cerebrovascular disease (stroke), increase in blood pressure (hypertension) and myocardial infarction are the precursor that causes CVD (Rastogi et al., 2016). CVD diseases involve the cardiovascular system that comprises of heart and veins. They are the main source of death universally, and its incidence is rising rapidly globally (Gaziano et al., 2010).

CVD diseases that often being suffered by people are heart failure, coronary heart disease (CHD), stroke, myocardial infarction, and hypertension. CHD and stroke, which responsible for 80% of CVD patients’ death, are caused by the lack of oxygen to the brain and heart. The accumulation of fatty deposits within the blood vessel causes the blocking of cerebral and coronary arteries hence narrows their pathway size (Roth et al., 2017). CHD contributes to almost 75% of worldwide death, which occurs both in low- and middle-income countries, due to few factors such as their socio-economy variations and risk factors, due to lifestyle modification (Gaziano et al., 2010).

Heart failure is caused by any functional or structural damage to ventricular filling or in the blood ejection (Klein et al., 2003). It is known as a leading cause of mortality in the western globe and tends to develop when cardiac injury or cardiac insult impairs the heart’s ability to maintain and pump tissue perfusion (Lymperopoulos et al., 2013). Hypertension, which is also known as a common CVD, is a crucial concern in health in diverse parts of the world. When the arterial pressure rises above 140/90 mm Hg., it is known as arterial hypertension (Malik et al., 2018).

Stroke is a heterogeneous disease, which includes the hemorrhage of cerebral and the pathogenic subtypes of ischemic stroke (Sacco et al., 2013). In the contribution of the major cause of death, stroke is one of the lead diseases. Among the stroke cases, 85% of them are ischemic stroke, and the other 15% is the hemorrhage stroke, which is also known as an intracerebral stroke (Woodruff et al., 2011). A clinical study shows that warfarin is being effective in preventing ischemic stroke in patients that are suffering from atrial fibrillation, which has reduced the risk of intracranial hemorrhage (Go et al., 2003). Hemorrhage is the rupture of the blood vessel that causes the blood to escape from its blood vessel. One of the main causes of stroke is due to blood coagulation. When the clotting blood accumulates in the blood vessel, the blood refuses to flow to the brain, and the brain tends to be lack of oxygen that leads to ischemic stroke. In this condition, an anticoagulant will avoid the blood from clotting and allow the ease of blood flow throughout the body and brain from the heart. Based on these regards, plants with anticoagulant activity will help treat cardiovascular diseases of these mechanisms.

Throughout human history, medicinal plants have always been used as medicine to treat various diseases. Almost 80% who live in developed countries are said to be depended on the practice of traditional medicine (Abdala et al., 2012). A report from the World Health Organization (WHO) comes out with a percentage of 80% of the global population tend to rely on traditional medicines. Most of the therapies use extracts and active compounds of the medicinal plant (Craig, 1999). Currently, there is a rise in medicinal plant consumption in the world, due to the proven effectiveness of medicinal plants, in curing certain diseases and claims that shows it is safe to be used (Perez Gutierrez and Baez, 2009). Medicinal plants play a major role in medication since the beginning of human civilization and also contribute to the manufacturing of drugs these days (Rastogi et al., 2016).

Asteraceae plant family is also used to be known as the Compositae plant family, is known as one of the largest plant families with thousands of plant species. Its large production as angiosperm phylogeny is in Asterideae. The Asteraceae plant family consists of 24,000 accepted species. It also has about 1,600 to 1,700 of its genera is distributed around the world, excluding Antarctica. This family is also known as a cosmopolitan family, as it has a great concentration of species in different areas such as temperate, cold-temperate, and subtropical. Asteraceae consists of three subfamilies; Asteroideae, Barnadesioideae, and Cichorioideae (Medeiros-Neves et al., 2018). The Lamiaceae plant family is also called a Labiatae family, which is often uttered as the mint family, and the plant family of flowering plants. They consist of shrubs or herbs which produce and release the aromatic smell, which consists of more than 3,000 species in the Lamiaceae plant family. The largest genera of Lamiaceae plant family are Salvia, Scutellaria, and Stachys (Cantino et al., 1992).

Plant species in Asteraceae and Lamiaceae family are being used to cured various diseases. Satureja species exhibits analgesic, antimicrobial, antiviral, antioxidant, antiproliferative, anti-inflammatory, and vasodilatory activities (Momtaz and Abdollahi, 2008). Meanwhile, *Crassocephalum crepidioides* (Benth.) S. Moore were used to cure epilepsy, indigestion, hepatotoxicity, swollen lips, tumor, and sleeping sickness (Bahar et al., 2016). Despite their variation in botanical features and traditional values, both Asteraceae and Lamiaceae plant family species exhibits mechanisms of improving cardiovascular disease. The seeds of *Gundelia tournefortti* L. (Asteraceae) are often used as pickles, and it is an effective diuretic (Çoruh et al., 2007). *Achillea millefolium* L., which is a plant species from the Asteraceae plant family, also exhibited diuretics effect in a hypertension group (De Souza et al., 2013). It is often found in Brazil and used as the Brazilian folk medicine, usually for kidney and heart diseases. *Emilia praetermissa* Milne-Redh. reduced hyperlipidemic conditions as an anticoagulant agent through a clinical study (Memariani et al., 2018). *Salvia miltiorrhiza* Bunge exerts the potential vasodilator effect on the cardiovascular system (Li et al., 1990). *S. miltiorrhiza* lowered whole blood viscosity, improved the peripheral circulation, and fastened erythropoiesis of erythrocytes (Lei and Chiou, 1986).

Medicinal plants are currently being used in developing a new hypertension drug, and one of them is *Marrubium vulgare* L. from Lamiaceae plant family. The crude oil of the plant was examined its hypotensive effect, and due to the presence of diterpenoids in the plant, it has potential cardiovascular activity, which was caused due to relaxation of vessels and reduces systolic blood pressure which are the mechanisms involved in hypertension (Bardai et al., 2001). The second example is *Cynara cardunculus* L. (syn. *Cynara scolymus* L.) from the Asteraceae plant family, which is also known as an artichoke. It is one of the oldest medicinal drugs for its cardiovascular effects. It exhibits a lipid-lowering effect and inhibits the biosynthesis of cholesterol (Fintelmaan, 1996). These drugs prove that medicinal plants can be developed into drugs or for as a treatment for therapeutic purposes. New drugs from the available medicinal plant can treat the disease more efficiently, as a safer and more efficient drug can be discovered in the future, which may benefit the patient (Bardai et al., 2001). Based on the traditional uses and the developed drug, it showed that the plant families have a high potential in alleviating cardiovascular diseases. Thus, an in-depth compilation of the activity and mechanism of the plant family on cardiovascular diseases needs to be examined.

**Figure 1:**
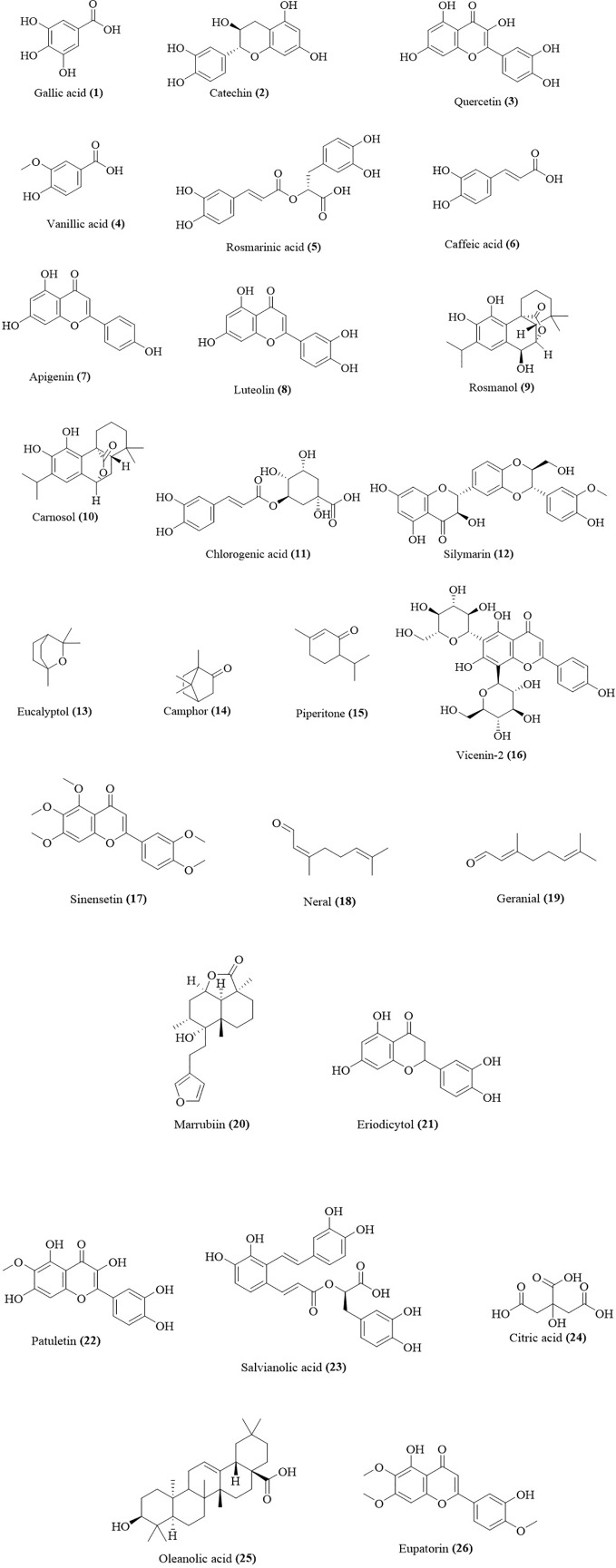
Chemical structures of phytochemicals with inhibitory activity on cardiovascular diseases from Asteraceae and Lamiaceae species.

A review on phytochemistry and pharmacological of medicinal plants related to cardiovascular diseases specifically on *Asteraceae* and *Lamiaceae* family were conducted by using searching engines such as Google Scholar, Scopus, ScienceDirect, ProQuest, Karger, and Molecule. The literature taken range from the year 1979 to 2018 and was evaluated and tabulated in this review. The keywords used during searching includes “cardiovascular diseases,” “coronary heart disease,” “stroke,” “myocardial infarction,” “hypertension,” “heart failure,” “antioxidant,” “anti-hyperlipidemia,” “antithrombotic,” “medicinal plants,” “plants,” “herbs,” “*in vivo,”* “*in vitro”* alone and in different combinations. This review specifically focuses on the identification and collection of the information on medicinal plants from the Asteraceae and Lamiaceae family, with proven *in vivo* or *in vitro* studies upon cardiovascular diseases. **Tables 1** and **2** show the plant species from Asteraceae and Lamiaceae family with their medicinal uses. **Tables 3** and **4** are on the mechanisms of the Asteraceae and Lamiaceae family plant species that are involved in cardiovascular diseases. Meanwhile, **Figure 1** is composed of chemical compounds derived from the two species that have potential as the lead drug in cardiovascular diseases.

**Table 1:** Medicinal uses of Asteraceae plant family species in cardiovascular diseases.

Plant Name	Country/Region	Common Name	Medicinal Uses	Part/s Used	Mode of Usage/Preparation	References
***Achillea arabica* Kotschy (syn. *Achillea biebersteinii* Hub.-Mor.)**	Mediterranean	Qaysoum	Hypolipidemic	Aerial parts	–	(Mais et al., 2016)
***Achillea millefolium* L**.	Europe, North America, Australia, and Asia	Mil-folhas	Diuretic and hypotensive actions	Aerial parts (leaves, stalks, and stems)	Aqueous extract	(De Souza et al., 2013)
***Achillea tenuifolia* Lam. (syn. *Achillea santolina* L.)**	Europe	Yarrow	Antioxidant	Aerial parts	–	(Ardestani and Yazdanparast, 2007)
***Ageratum conyzoides* (L.) L**.	Brazil	Billygoat-weed	Hypolipidemic	Leaf, stem, and root	–	(Atawodi et al., 2017)
***Anthemis melampodina* subsp. *deserti* (Boiss.) Eig (syn. *Anthemis deserti* Boiss)**	Saudi Arabia	–	Antioxidant	Whole plant	Aqueous extract	(Shahat et al., 2014)
***Artemisia absinthium* L**.	Europe, North America, and Asia	Wormwood	Antioxidant	Aerial parts (leaves, stalks, and stems)	Methanolic extract	(Bora and Sharma, 2011)
***Artemisia campestris* L**.	Eastern Morocco	–	Antihypertensive and vasorelaxant	Aerial parts	–	(Dib et al., 2017)
***Baccharis trimera* (Less.) DC**	South America	Carqueja	Vasorelaxant	Whole plants	Infusions, decoctions, and tinctures of its aerial parts	(Sabir et al., 2017)
***Bidens pilosa* L**.	South America	Spanish needles, beggar’s ticks, devil’s needles	Antihypertensive, vasodilation	Leaf	Dry powder, decoction, maceration or tincture	(Dimo et al., 2001)
***Chamaemelum nobile* (L.) All**.	Roman chamomile	–	Hypotensive and diuretics	Whole plant	–	(Zeggwagh et al., 2009)
***Chromolaena odorata* (L.) *R. M. King and H. Rob*. **	South and Central America, India	Ahihia eliza or Siam Weed	Anti-hyperlipidemic	Leaves	Fresh leaves or decoction	(Ikewuchi and Ikewuchi, 2011)
***Chrysanthemum* x *morifolium* Ramat. Hemsl**.	Japan	Chrysanthemum	Vasodilation	Flowers	Extract	(Gao et al., 2016)
***Crassocephalum crepidioides* (Benth.) S. Moore**	Africa	Okinawa Spinach, Red flower	Antioxidant and anti-hyperlipidemic	Aerial Parts (leaves, stalks, and stems)	Maceration	(Bahar et al., 2016)
***Cynara cardunculus* L. (syn. *Cynara scolymus* L.)**	Mediterranean	Global artichoke	Hypolipidemic	Leaf	Aqueous extract	(Mocelin et al., 2016)
***Eclipta prostrata* (L.) L**.	India, Nepal, China, and Brazil	False daisy	Hypolipidemic	Leaves	Herb or plant juice taken orally	(Dhandapani, 2007)
***Emilia praetermissa* Milne-Redh**	Sierra Leone and Nigeria	Kipo or Koyagipo	Lipid-lowering effect	Leaves	Orally consumed as fresh salads or cooked. Maceration for improving heart conditions.	(Ngozi et al., 2013)
***Erigeron canadensis* L**.	North America and Central America	Conyza canadensis	Antithrombotic	Flowering parts	Raw material	(Pawlaczyk et al., 2011)
***Flaveria bidentis* (L.) Kuntze**	South America	Coastal plain yellowtops	Anticoagulant	Leaves	–	(Guglielmone et al., 2002)
***Gundelia tournefortti* L**.	South America	Kuub	Hypolipidemic	Seeds	Oil extract	(Sharaf and Ali, 2004)
***Gymnanthemum amygdalinum* (Delile) Sch. Bip. (syn. *Vernonia amygdalina* Delile)**	Africa	Bitter leaf	Hypolipidemia and antioxidant	Leaf	Orally consumed	(Audu et al., 2012)
***Helichrysum leucocephalum* Ausfeld**	Eurasia, Africa, and Australi	Curry plant or Italian strawflower	Antioxidant	Aerial parts (leaves, stalks, and stems)	Extract	(Goldansaz et al., 2018)
***Inula racemosa* Hook F**.	India, Asian	Pushkarmool	Hypotensive, antihyperlipidemic and antioxidant	Roots	Administered orally for rheumatic pains	(Mangathayaru et al., 2009)
***Laggera decurrens* (Vahl) Hepper and J. R. I. Wood**.	Africa	Fwimba	Antioxidant	Aerial parts	Traditional herb	(Mothana et al., 2011)
***Launaea intybacea* (Jacq.) Beauverd (syn. *Lactuca runcinata* DC.)**	India	Lettuce	Anti-hyperlipidemic	Whole plant	Ethanolic extract	(Devi and Muthu, 2015)
***Leuzea carthamoides* Willd. DC**.	Russian	Maral root	Antiplatelet	Leaves	–	(Koleckar et al., 2008)
***Pectis brevipedunculata* Sch. Bip**	Brazil, America	Lemongrass	Vasorelaxant	Aerial parts	Orally consumed as tea, juice drinks or spices	(Pereira et al., 2013)
***Senecio nutans* Sch. Bip**	South America	Senecio graveolens	Hypotensive and antihypertensive effect	Braches and leaves	Extract	(Cifuentes et al., 2016)
***Senecio ovatus* subsp. *stabianus* (Lacaita) Greuter (syn. *Senecio stabianus* Lacaita)**	Italy	–	Antioxidant	Aerial parts	Extract	(Tundis et al., 2012)
***Silybum marianum* (L.) Gaertn**.	Mediterranean region of Europe	Milk thistle, Mary thistle	Antioxidant, anti-cholesterolemia	Seed	Extract	(Taleb et al., 2018)
***Solidago chilensis* eyen**	Southern America	Arnica-do-brazil	Hypolipidemic and antioxidant	Aerial parts	–	(Schneider et al., 2015)
***Sphaeranthus indicus* L**.	India	Gorakhmundi	Antihyperlipidemic	Flower	Extract	(Pande and Dubey, 2009)
***Tagetes erecta* L. (syn. *Tagetes patula* L.)**	France	Jafri	Hypotensive	Roots (nematocidal thiophenes)	Perfume	(Saleem et al., 2004)
***Tanacetum vulgare* L**.	Europe and Asia	Tansy	Diuretics	Leaves	–	(Lahlou et al., 2007)
***Tridax procumbens* (L.) L**.	India	Ghamra or coat buttons	Antithrombotic	Leaves	–	(Naqash and Nazeer, 2011)
***Vernonia elaeagnifolia* DC**	Asia and Europe	Toran vel, curtain creeper	Anti-hyperlipidemic	Leaf	Aqueous extract	(Khandekar et al., 2015; Sultana et al., 2017)

**Table 2:** Medicinal uses of Lamiaceae plant family species in cardiovascular diseases.

Plant Name		Country/Region	Common Name	Medicinal Uses	Part/s Used	Mode of Usage/Preparation	References
***Agastache mexicana* (Kunth.) Lint. and Epling**.		Mexico	Mexican giant hyssop	Vasorelaxant	Aerial parts	–	(Hernandez-Abreu et al., 2013)
***Ajuga integrifolia* Buch.-Ham. ex D. Don (syn. *Ajuga remota* Benth.)**		Ethiopia	Armagusa	Diuretics	Leaves	Methanolic extract	(Hailu and Engidawork, 2014)
***Ajuga iva* (L.) Schreb**.		Mediterranean	Southern Bugle	Antioxidant and hypolipidemic	Whole plant	Aqueous extract	(Taleb-Senouci et al., 2009)
***Ballota glandulosissima* Hub. -Mor. and Patzak**		Turkey	Horehound	Antioxidant	Aerial parts	External use or aerial parts used internally	(Citoglu et al., 2004)
***Clerodendrum volubile* P.Beauv**		Nigeria	Marugbo	Antihyperlipidemic	Leaves	Leaf extract (dried and blended fresh leaves)	(Akinpelu et al., 2016)
***Clinopodium vulgare* L. (syn. *Calamintha vulgaris* (L.) Druce)**		Pakistan	Wild basil	Antihypertensive and vasodilation	Aerial parts	Crude extract and methanolic extract	(Khan et al., 2018)
***Dracocephalum moldavica* L**.		Central Asia	Moldavian dragonhead	Antioxidant and cardioprotective	Aerial parts	Oral consumption as food or tea	(Jiang et al., 2014)
***Isodon rugosus* (Wall. ex Benth) Codd**		Pakistan	Wall. ex Benth	Vasorelaxant and antioxidant	Aerial parts	–	(Janbaz et al., 2014)
***Lagenaria siceraria* (Mol.) Standl**		African	Long melon, New Guinea bean and Tasmania bean	Cardioprotective, antihyperlipidemic, and diuretic activities.	Fruit	Fruit powder	(Mali et al., 2012)
***Lallemantia royleana* (Benth.) Benth**.		Iran	Balangu	Hypolipidemic	Seed	Oral consumption	(Ghannadi et al., 2015)
***Lavandula angustifolia* Mill**.		Iran	Ostokhoddus	Antioxidant	Aerial parts	Essential oil	(Ziaee et al., 2015)
***Lavandula stoechas* L**.		Morocco	French lavender	Antioxidant	Aerial parts	–	(Ezzoubi et al., 2014)
***Leonotis leonurus* (L.) R. Br**.		South Africa	Lion’s tail/Wild dagga	Anticoagulant and antiplatelet	Leaves	Organic extract	(Mnonopi et al., 2011)
***Leonurus cardiaca* L**.		Europe	Matthiolus	Antiarrhythmic	Aerial parts	–	(Ritter et al., 2010)
***Lepechinia caulescens* (Ortega) Epling**		Mexico	Island pitchersage	Antihypertensive and vasorelaxant	Aerial parts	Oral beverage or tea	(Estrada-Soto et al., 2012)
***Leucas aspera* (Willd.) Link**		India	Thumbai	Antihyperlipedimia	Leaf	Ethanolic extract	(Kumar, 2016)
***Melissa officinalis* L**.		Anatolia and Mediterranean	Lemon balm	Vasodilation	Leaves	Consumed orally as tea	(Ersoy et al., 2008)
***Micromeria macrosiphon* Coss. (syn. *Satureja macrosiphon* (Coss.) Maire)**		Morocco	Maire	Antioxidant	Aerial parts	Extract	(Amiri, 2011)
***Origanum vulgare* L**.		Europe, North Africa, America	Oregano	Antioxidant	Whole plants	Traditional medicine	(Zhang et al., 2014)
***Orthosiphon aristatus* (Blume) Miq. (syn. *Orthosiphon stamineus* Benth.)**		Indonesia, Asia	Kumis kucing, Misai kusing	Antihypertensive and vasorelaxant	Leaves, whole plant	Water decoction, extract	(Matsubara et al., 1999; Akowuah et al., 2005; Yam et al., 2016; Yam et al., 2018)
***Phlomoides bracteosa* (Royle ex Benth.) Kamelin and Makhm. (syn. *Phlomis bracteosa* (Royle ex Benth.) Kamelin and Makhm.)**		Pakistan	Jerusalem sage	Vasodilation	Whole plant	Methanolic extract	(Khan et al., 2012)
***Plectranthus hadiensis* (Forssk.) Schweinf. ex Sprenger (syn. *Coleus forskohlii* Willd.)**		India	Plectranthus barbatus	Antihypertensive and vasodilation	Whole plant	Ethanolic extract	(Dubey et al., 1981)
***Plectranthus monostachyus* (P. Beauv.) B. J. Pollard (syn. *Solenostemon monostachyus* (P. Beauv.) Briq.)**		Ivory coast	Coleus	Antioxidant and antihypertensive	Leaves	Ethanolic extract	(Fidele et al., 2012)
***Pogostemon elsholtzioides* Benth**.		Eastern Himalaya	Nakhrang sheng	Vasorelaxant and antihypertensive	Leaves	Leaf decoction	(Shiva Kumar et al., 2017)
***Prunella vulgaris* L**.		–	Self-heal	Antihyperlipidemic and antioxidant	Rhizome/root	Hydroalcoholic and aqueous extract	(Zargar et al., 2017)
***Rosmarinus officinalis* L**.		Mediterranean countries	–	Anti-hypercholesterolemic	Leaves	Water decoction	(Belmouhoub et al., 2017)
***Salvia miltiorrhiza* Bunge**		China and Japan	Danshen	Antithrombosis	Root	Oral consumption of dried root	(Fan et al., 2010)
***Salvia ofﬁcinalis* L**.		Mediterranean	Sage	Antioxidant	Shoots	Raw material, essential oils or extract	(Santos-Gomes et al., 2002)
***Salvia scutellarioides* Kunth**		Colombia	Mastranto	Antihypertensive and diuretic effects	Leaves and stem	Aqueous extract	(Ramirez et al., 2006)
***Satureja cuneifolia* Ten. (syn. *Satureja obovata* Lag.)**		Lanjoran	Thin savory	Vasodilation and vasorelaxant	Whole plant	Extract	(De Rojas et al., 1999)
***Sideritis raeseri* Boiss. and Heldr**.		Mediterranean	Ironwort, mountain tea, and shepherd’s tea	Hypotension and vasodilatation	Aerial parts	–	(Kitic et al., 2012)
***Teucrium polium* Linn**.		South-western Asia and Europe	Felty germander	Hypolipidemic	Aerial parts	Aqueous extract	(Rasekh et al., 2001)
***Thymus dreatensis* Batt. (syn. *Thymus atlanticus* (Ball) Pau)**		Morocco	Thyme, German thyme	Antihyperlipidemic and anticoagulant	Whole plant	Extract	(Ramchoun et al., 2012)
***Thymus saturejoides* Coss**.		Morocco	Thyme borneol	Antioxidant	–	–	(Khouya et al., 2015)
***Thymus serpyllum* Linn**.		Europe and North America	Breckland thyme	Antihypertensive	Whole plant	Culinary herb	(Katalinic et al., 2006)
***Thymus zygis L*. **		Morocco	Thyme, sauce thyme	Anticoagulant	Whole plant	Extract	(Ocana and Reglero, 2012; Khouya et al., 2015)
***Vitex megapotamica* (Spreng.) Moldenke**		South America	Forest olive	Antihyperlipidemic	Leaves	Extract	(Pires et al., 2018)
***Ziziphora clinopodioides* Lam**.		China	Blue mint bush	Vasodilation and antihypertensive	Whole plant	Decoction of whole plant	(Senejoux et al., 2010)

**Table 3:** Mechanism of action of Asteraceae family plant species.

Plant Name	Parts Used	Isolated Compound/Extract		Class	*In Vivo/In Vitro*	Mechanism of Action	References
***Achillea arabica* Kotschy (syn. *Achillea biebersteinii* Hub.-Mor.)**	Aerial parts		Ethanolic extract	Sesquiterpene lactones, polyphenols, and flavonoids.	*In vivo*	The extract at a dose of 400 mg/kg showed a significant decrease in the levels of serum cholesterol, triglycerides, and LDL. It also significantly decreased hepatic total cholesterol and triglycerides.	(Mais et al., 2016)
***A. millefolium* L**.	Aerial parts (leaves, stalks, and stems)	Hydroethanolic extract (HEAM), Dichloromethane (DCM), and armetin		Flavonoid	*In vivo*	With the dose of 300 mg/kg of HEAM, it increased the diuresis around 30–60% between 4 and 8 h after administration. The diuresis effect decreased systemic vascular resistance. The extract also reduced blood volume and cardiac output. A single dose of HEAM (100 mg/kg), which was administered to the rats 3 h before measurement, showed a lower MAP reading by 13 ± 1 mm Hg. Increasing the dose to 300 mg/kg, decreased MAP by 14 ± 3 mm Hg.	(De Souza et al., 2013)
***A. tenuifolia* Lam. (syn. *Achillea santolina* L.)**	Aerial		Hydroalcoholic extract (ASE)	Phenol and flavonoid	*In vitro*	At high concentrations (200 and 400 kg/mL), ASE suppressed lipid oxidation, by extending the lag phase and reducing the propagation rate. It reflects a typical characteristic of a chain-breaking antioxidant, similar to that of known antioxidants.	(Ardestani and Yazdanparast, 2007)
***Ageratum conyzoides* (L.) L**.	Leaf, stem, and root		Methanolic extract	Alkaloids, carbohydrate, cardiac glycosides, flavonoids, saponins, tannins, steroids, and triterpenes	*In vivo*	The leaves and stem extracts (100 mg/kg) lowered total cholesterol, LDL-C, and triglycerides level.	(Atawodi et al., 2017)
***A. melampodina* subsp. *deserti* (Boiss.) Eig (syn. *Anthemis deserti* Boiss)**	Whole plant		Methanolic extract	Flavonoid	*In vitro*	The extract exhibited antioxidant capacity at 400 µg/mL. All concentrations of the extract tested possessed radical scavenging activity. Higher concentrations of the extract showed similar activity as standards.	(Shahat et al., 2014)
***Artemisia absinthium* L**.	Aerial parts		Quercetin **(3)**, rutin, isoquercitrin, quercitin- 3-*O*-β-d-glucoside, glucoside, chlorogenic, syringic, coumaric, salicylic, and vanillic acids **(4)**.	Flavonoids, flavonoid glycosides, phenolic acid	*In vitro*	The extract showed a significant (p < 0.05) activity at the dose of 100 μg/mL in the scavenging of superoxide anion radical. Pre-treatment of ischemic brain mouse with the extract significantly (p < 0.05) decreased the elevated TBARS concentration in brain mitochondrial and supernatant fractions as compared to the control group. Reducing power of the extract data suggests that it contributes significantly to the observed antioxidant effect.	(Bora and Sharma, 2011)
***Artemisia campestris* L**.	Aerial parts		3,5-dicaffeoylquinic (isochlorogenic A) acid, 5-caffeoylquinic (chlorogenic) acid, and vicenin-2 **(16)**.	Flavonoids	*In vivo*	The extract at the dose of 150 mg/kg/day prevented hypertension on hypertensive rats and reduced SBP from 172 mm Hg to 144 mm Hg. At the dose of 40 mg/kg, the extract reduced SBP, DBP, and MAP, without affecting the heart rate. The extract (10^−2^–2 mg/mL) relaxed the pre-contracted aorta by 95.8 ± 1.3%.	(Dib et al., 2017)
***Baccharis trimera* (Less.) DC**	Whole plant		Aqueous extracts (rutin and quercetin **(3)**)	Flavonoids and terpenes	*In vitro*	The aqueous extract showed higher efficiency in eliminating DPPH radical with an IC_50_ value of 415 ± 12.1 μg/mL. The extract was capable of reducing deoxyribose damage at all concentrations by its ability to chelate iron by greater than 50% at the extract concentration of 100 μg/mL.	(Sabir et al., 2017)
***Bidens pilosa* L**.	Leaf		Aqueous and methylene chloride extracts	Flavonoids, alkaloids, saponins, phenyl acetylenes, and terpenes	*In vivo*	Aqueous extract (150 or 350 mg/kg) and methylene chloride extract (150 mg/kg or 300 mg/kg) of *B. pilosa* completely blocked the elevation of blood pressure in fructose-treated rats and provoked a decline toward control values. The extracts reversed the increase in SBP.	(Dimo et al., 2001)
***Chamaemelum nobile* (L.) All**.	Whole plant		Aqueous extract	–	*In vitro* and *in vivo*	Single oral administration of the extract (140 mg/kg) produced a significant reduction in SBP. Daily oral administration of the extract (140 mg/kg) during three weeks, produced a significant reduction in SBP in day eight of treatment. The extract produced a significant increase in urinary output and electrolytes excretion.	(Zeggwagh et al., 2009)
***Chromolaena odorata* (L.) *R. M. King* and *H. Rob*. **	Leaves		Aqueous extract	–	*In vivo*	100 mg/kg of aqueous extract reduced triglycerides, LDL, VLDL, non-HDL, and total cholesterol. The HDL-C level of the treated animals was significantly higher.	(Ikewuchi and Ikewuchi, 2011)
***Chrysanthemum* x *morifolium* Ramat. Hemsl**.	Flower		*C. morifolium* extract (CME)	Polyphenols	*In vivo*	Polyphenol-rich CME alleviated hypertensive cardiac hypertrophy in rats through the reduction of blood pressure. Administration of CME at the dose of 75–150 mg/kg for four weeks lowered the SBP.	(Gao et al., 2016)
***C. crepidioides* (Benth.) S. Moore**	Aerial parts		Aerial methanolic extract, coumarin, and reducing sugar	Alkaloids, glycosides, cardiac steroids, tannins, flavonoids, saponins, and glycosides	*In vitro* and *in vivo*	Increasing concentration of the methanolic extract increased its DPPH radical scavenging activity. The Wistar albino rats were administered with plant extract (150 and 300 mg/kg/day) orally. It significantly reduced the serum total cholesterol, triglycerides, LDL-C, VLDL-C levels, and significantly increased serum HDL-C level compared with a positive control group. The dose of 300 mg/kg showed significant (p < 0.01) antihyperlipidemic activity compared with the positive control group.	(Bahar et al., 2016)
***C. cardunculus* L. (syn. *Cynara scolymus* L.)**	Leaf		Quercetin **(3)**	Phenols and flavonoids	*In vitro* and *in vivo*	The extract exhibited a free radical scavenging effect. Hyperlipidemic rat administered with the extract of 150 to 600 mg/kg decreased triglyceride and LDL-C levels. It also reduced HMG-CoA reductase enzyme activity, hence reduced the formation of VLDL from the liver.	(Mocelin et al., 2016)
***Eclipta prostrata* (L.) L**.	Leaves		Leaf extract	Alkaloids, phytosterols, flavonoids, saponins, tannins, sugar	*In vivo*	The extract reduced total cholesterol, triglyceride, protein, and increased HDL-C. The extract (100 and 200 mg/kg) showed a significant hypolipidemic effect.	(Dhandapani, 2007)
***E. praetermissa* Milne-Redh**	Leaves	Aqueous extract		Tannins, cardiac glycosides, flavonoids, terpenoids	*In vivo*	The aqueous extract significantly reduced the triglyceride level (47.80 ± 4.75 mg/dl to 37.22 ± 2.18 mg/dl) at a dose of 200 mg/kg after 2 h. The level of HDL was significantly increased (48.44%) by 400 mg/kg of the extract. The extract (100 mg/kg) caused significant reductions in LDL level when it was administered concomitantly with 15 and 30 mg/kg atorvastatin, respectively.	(Ngozi et al., 2013)
***Erigeron canadensis* L**.	Flowering parts		Plant extract (polysaccharide- polyphenolic)	Flavonoids and tannins	*In vitro* *In vivo*	The extract inhibited thrombin and factor Xa amidolytic activities in the presence of antithrombin. The plant preparation inhibits plasma clot formation in aPTT at the concentration as low as 390 μg/mL of standardized human blood plasma, and in PT test at the concentration of 1.56 mg/mL. The strong anticoagulant effect was observed after 40 min after the administration, with the clotting time almost three times longer than control measurement.	(Pawlaczyk et al., 2011)
***Flaveria bidentis* (L.) Kuntze**	Leaves		Quercetin 3-acetyl-7,3′,4′-trisulfate (ATS) and quercetin 3,7,3′,4′-tetrasulfate (QTS)	Flavonoids	*In vitro*	QTS has higher activity than ATS in activating heparin cofactor II (HCII), indicating that these flavonoids act as agonists of this inhibitor. The flavonoids also increased PT with a concentration of 1 mM of QTS (25.2 ± 0.8 s, p < 0.01) and ATS (22.2 ± 0.7 s, p < 0.04). It also prolonged aPTT at the concentration of 112 ± 11 and 53 ± 2, respectively.	(Guglielmone et al., 2002)
***G. tournefortti* L**.	Seeds		Tyramine	Saponin and alkaloid	*In vivo*	The oil extract (90 mg/kg) possessed a hypolipidemic effect by reducing plasma total lipid, total cholesterol, VLDL-cholesterol, LDL-cholesterol, and atherogenic indices. It also increased the HDL value and reduced the total cholesterol level in the liver.	(Sharaf and Ali, 2004)
***Gymnanthemum amygdalinum* (Delile) Sch. Bip. (syn. *Vernonia amygdalina* Delile)**	Leaf		Aqueous extract	Flavonoids and phenolics	*In vivo*	The extract caused a decrease in plasma total cholesterol, LDL, triacylglycerol, and VLDL and an increase in plasma HDL-C concentration of hyperlipidemic animals.	(Audu et al., 2012)
***H. leucocephalum* Ausfeld**	Aerial parts (leaves, stalks, and stems)		Chalcones, phthalides, α-pyron derivatives, essential oils, volatiles, and fatty acids	Phenol, terpenoids, and flavonoids	*In vitro* and *In vivo*	The extracts exhibited scavenging activity towards DPPH radicals.	(Goldansaz et al., 2018)
***Inula racemosa* Hook F**.	Roots		Alcohol extract (essential oil of the roots), phenyl acetonitrile and phenyl ethanol	Sesquiterpenes and phenolics	*In vivo*	IrA decreased total cholesterol, triglycerides, LDL-C, and the atherogenic index, and increased HDL-C compared with the positive control. It also reduced GSH in both the tested tissues, levels of endogenous antioxidants SOD and GPX in the heart. It inhibited lipid peroxidation, and reduced lipid uptake, resulting in a reduction of fatty streak formation, *via* decreased foam cell formation.	(Mangathayaru et al., 2009)
***L. decurrens* (Vahl) Hepper and J. R. I.Wood**.	Aerial parts		Essential oil (3-methoxythymoquinone, thymol, and carvacrol)	Monoterpenes, Phenols	*In vitro*	The extract (500 µg/mL) exhibited high antioxidant activity (91%) by scavenging DPPH.	(Mothana et al., 2011)
***Launaea intybacea* (Jacq.) Beauverd (syn. *Lactuca runcinata* DC.)**	Whole plant		Ethanolic extract	–	*In vivo*	At a dose of 200 mg/kg, the extract reduced the level of plasma total cholesterol, ester cholesterol, free cholesterol, free fatty acid phospholipids, and triglycerides in comparison with AD rat. Whereby at a dose of 400 mg/kg, the extract increased the rats’ HDL-C level. The reduction of the plasma lipid and lipoprotein profile was due to the presence of phenolic and flavonoids compounds. HDL reversing cholesterol transport, inhibiting the oxidation of LDL and neutralizing the atherogenic effects of oxidized LDL.	(Devi and Muthu, 2015)
***Leuzea carthamoides* Willd. DC**.	Leaves		Eriodictyol **(21)** and patuletin **(22)**	Flavonoid	*In vitro*	Eriodictyol **(21)** and patuletin **(22)** exhibited antiplatelet activity. They inhibited COL- and AA-induced platelet aggregation.	(Koleckar et al., 2008)
***Pectis brevipedunculata* Sch. Bip**	Aerial parts		Essential oil (Citral, geranial **(19)**, limonene, and α-pinene)	Monoterpene compounds, hydrocarbons, sesquiterpenes, alcohols and aldehydes	*In vitro*	The essential oil caused vasorelaxation activity. The citral possessed vasodilator activity towards KCl-contracted aorta. Citral attenuated the contracture induced by Ca^2+^ in the depolarized aorta. EOPB and citral elicited vasorelaxation on thoracic aorta by affecting the NO/cyclic GMP pathway and the calcium influx through voltage-dependent L-type Ca^2+^ channels.	(Pereira et al., 2013)
***Senecio nutans* Sch. Bip**	Branches and leaves	Hydroalcoholic extract, dihydroeuparin,p-hydroxy acetophenone		Terpenes and flavonoids	*In vivo*	The plant extract (40 mg/kg) caused a reduction in SBP and DBP by 23% and 35%, respectively. The extract also decreased MAP and heart rate by intravenous (IV) route administration, in addition to prolonged dilatation time.	(Cifuentes et al., 2016)
***S. ovatus* subsp. *stabianus* (Lacaita) Greuter (syn. *Senecio stabianus* Lacaita)**	Aerial parts		Plant extract	Phenol and flavonoid	*In vitro*	Ethyl acetate extract showed the highest activity with IC_50_ values of 35.5 and 32.7 mg/mL on the DPPH test and ABTS test, respectively.	(Tundis et al., 2012)
***Silybum marianum* (L.) Gaertn**.	Seeds	Silybin (the main component of Silymarin mixture)		Polyphenols	*In vivo*	Silymarin **(12)** acts as a free radical scavenger such as (OH, O_2_) and it enhances the antioxidant enzymes CAT, SOD, and GPx. Thus, it increased the antioxidant cell defense and the activity of the mitochondrial enzyme. It caused activation of Nrf2 and inhibited NF-kB, and expressions of eNOS and MAPK (ERK1, 2, JNK). It activated the ribosome and increased protein synthesis to regenerate cardiovascular tissues. It also scavenged free radicles in the cytoplasm and increased ribosomal RNA synthesis. A dose of 200 mg/kg of silymarin **(12)** reduced the ROS level of rats when given intraperitoneally. 100 mg/kg of silymarin **(12)** increased HDL-cholesterol and decreased liver cholesterol of hypercholesterolemic rats.	(Taleb et al., 2018)
***Solidago chilensis* Meyen**	Aerial parts		Hydroalcoholic extract	Flavonoids	*In vitro* *In vivo*	The extract exhibited antioxidant properties with an IC_50_ value of 59.12 ± 3.14 µg/mL. The extract at the dose of 125, 250, or 500 mg/kg decreased the total cholesterol of rats.	(Schneider et al., 2015)
***Sphaeranthus indicus* L**.	Flower		Ethanolic extract	Tannin	*In vivo*	The extract of dose 500 mg/kg/day given in rat orally for eight days caused a decrease in body weight, total cholesterol, triglycerides, LDL, and VLDL. It showed a rise in HDL level resembling its use in atherosclerosis conditions.	(Pande and Dubey, 2009)
***Tagetes erecta* L. (syn. *Tagetes patula* L.)**	Roots		Citric acid **(24)**, dimethyl citrate, and malic acid	Tricarboxylic acid	*In vivo*	The citric acid **(24)** reduced the blood pressure of normotensive rats in a dose-dependent manner. It caused 17–21% and 32–35% fall in MABP at the corresponding doses of 3 and 30 mg/kg, respectively.	(Saleem et al., 2004)
***T. vulgare* L**.	Leaves		Plant extract	–	*In vivo*	Administration of 10 mg/kg of the leaf extract caused an increase in urine output. The levels of Na^+^ and K^+^ in the urine increased, but the plasma Na^+^ and K^+^ were not affected in this activity.	(Lahlou et al., 2007)
***Tridax procumbens* (L.) L**.	Leaves		Extract	Sulfated polysaccharide	*In vitro*	The sulfated polysaccharides prolonged aPTT (113 s) at a dose of 100 μg/mL, which was approximately 4.0-fold compared with the saline group.	(Naqash and Nazeer, 2011)
***Vernonia elaeagnifolia* DC**.	Leaf		Ethanolic extract	Flavonoids, phenolic compounds, tannins, terpenoids, phytosterols, alkaloids, and coumarins	*In vivo*	The extract restored the levels of LDL and HDL cholesterol of albino rabbits.	(Sultana et al., 2017)

**Table 4:** Mechanism of action of Lamiaceae family plant species.

Plant Name	Parts Used	Isolated Compound/Extract	Class	*In Vivo/In Vitro*	Mechanism of Action	References
***Agastache mexicana* (Kunth.) Lint. and Epling**.	Aerial parts	Acacetin, oleanolic acid **(25)**, and ursolic acid	–	*In vivo*	Each hypertensive mouse received an intragastric dose of ursolic acid (50 mg/kg). It inhibited vasoconstriction induced by KCl and noradrenaline bitartrate (NA) in endothelium-denuded aortic rings, and also inhibited the concentration-response contraction of NA in a nonparallel manner and depressed its maximal response. The extract at the dose of 112, 200, and 625 µg/mL possessed Ca_2+_entry blocking activity.	(Hernandez-Abreu et al., 2013)
***Ajuga integrifolia* Buch.-Ham. ex D. Don (syn. *Ajuga remota* Benth.)**	Leaves	phenolic compounds, tannins, saponins, flavonoids, terpenoids, steroids, and cardiac glycosides	–	*In vivo*	80% methanolic extract produced significant diuresis (p < 0.01), while the aqueous extract had shown diuresis both at the middle (p < 0.01) and higher (p < 0.01) doses by the end of the fifth hour of administration.	(Hailu and Engidawork, 2014)
***Ajuga iva* (L.) Schreb**.	Whole plant	Aqueous extract	Ecdysone, terpenoid, flavonoid	*In vitro*	The extract (1 mg/mL) reduced plasma cholesterol and triacylglycerol. It also reduced TBARS concentration, the lipid peroxidation product. Product concentration was reduced	(Taleb-Senouci et al., 2009)
***Ballota glandulosissima* Hub. -Mor. and Patzak**	Aerial parts	kumatakenin, pachypodol, 5-hydroxy-7,3′,4′-trimethoxyﬂavone, velutin, corymbosin, retusine	Flavonoid	*In vivo*	The extract inhibited lipid peroxidation with an IC_50_ value of 12 to 20 mg/mL.	(Sever, 2000; Citoglu et al., 2004)
***Clerodendrum volubile* P. Beauv**	Leaf	Leaf extract (alkaloids, saponins, tannins, flavonoids, steroids, and cardiac glycosides)	–	*In vivo*	The extract (250 and 500 mg/kg) significantly lowered the total cholesterol, LDL, VLDL, and triglycerides, and increased HDL level dose-dependently, in both phyto-preventive and curative animals.	(Akinpelu et al., 2016)
***C. vulgare* L. (syn. *C. vulgaris* (L.) Druce)**	Aerial parts	Crude extract	Phenolic and flavonoid	*In vivo* and *in vitro*	The extract and fractions showed an antihypertensive effect at doses of 10 and 30 mg/kg, respectively, by reducing the MAP of hypertensive rats.	(Khan et al., 2018)
***Dracocephalum moldavica* L**.	Aerial parts	Plant extract	Flavonoid	*In vitro*	The ﬂavonoids fraction, at the concentration of more than 70 mg/L, exhibited a higher DPPH radical scavenging activity than vitamin E. The ﬂavonoids fraction also possessed scavenging activity on DPPH, hydroxyl radicals, and superoxide anion radicals.	(Jiang et al., 2014)
***Isodon rugosus* (Wall. ex Benth) Codd**	Aerial parts	Crude extract	–	*In vivo* and *in vitro*	The crude extract (0.01–0.3 mg/mL) possessed a relaxant effect on isolated rabbit jejunum, trachea, and aorta preparations. The mechanism of action was assumed to be by the Ca^2+^ channel blockade.	(Janbaz et al., 2014)
***Lagenaria siceraria* (Mol.) Standl**	Fruit	C-glycosides	Flavone	*In vivo*	*L*-NAME was used to induce hypertension in rats. Concomitant treatment of *L. siceraria* fruit powder (LS) and *L*- NAME for 28 days reduced cholesterol level but did not reduce triglycerides levels. It also reduced SBP, DBP, and MABP significantly. LS, as well as L-arginine treatment, produced significant (p <0.001) attenuation of the hypertensive effect of *L*- NAME.	(Mali et al., 2012)
***Lallemantia royleana* Benth. in Wall**.	Seeds	Oleic, linoleic, and linolenic acid content	–	*In vivo*	Administration of the seeds for 12 weeks decreased (p < 0.05) the rabbit’s total serum cholesterol and triglycerides. It also significantly decreased LDL-C and HDL-C of the hypercholesterolemic group (p < 0.05).	(Ghannadi et al., 2015)
***Lavandula angustifolia* Mill**.	Aerial parts	Essential oil (linalool, linalyl acetate camphor **(14)**, 1,8-cineol, luteolin **(8)**, triterpenoids like ursolic are and coumarin)	Mono and sesquiterpenes, flavonoids	*In vivo*	The essential oil (10 mg/kg) significantly decreased heart to body weight ratio (p < 0.001). Treatment with 10 and 20 mg/kg of essential oil demonstrated a profound reduction (p < 0.001) in the ST-segment elevation.	(Ziaee et al., 2015)
***Lavandula stoechas* L**.	Aerial parts	Ethanolic extract	–	*In vitro*	The extract exhibited antioxidant activity by scavenging DPPH with an IC_50_ value of 1.2 μg/mL while the IC_50_ value of the reference standard, BHT was 0.2 μg/mL.	(Ezzoubi et al., 2014)
***Leonotis leonurus* (L.) R. Br**.	Leaves	Marrubiin **(20)** and organic extract	Diterpenoids	*In vivo* and *in vitro*	The extract (25–2,000 µg/mL) and marrubiin **(20)** (1.25–100 µg/mL) inhibited platelet aggregation by suppressing the binding of fibrinogen to the surface receptor GP2b/3a. They also inhibited collagen and thrombin-induced calcium mobilization. Diterpenoids inhibited the extracellular receptor kinase (ERK) 1/2 signaling pathway.	(Mnonopi et al., 2011)
***Leonurus cardiaca* L**.	Aerial parts	Leonurus cardiaca refined extract (LCRE) Cardioactive lavandulifolioside and verbascoside	Phenylethanoid glycosides	*In vitro* and *in vivo*	LCRE at the dose of 1.0 to 2.0 mg/mL was infused intracoronary for 10 min before mapping its epicardial potential. It reduced the left ventricular pressure in a dose-dependent manner and elevated the relative coronary flow.	(Ritter et al., 2010)
***Lepechinia caulescens* (Ortega) Epling**	Aerial parts	Methanolic extract (ursolic acid, terpinene- 4-ol, salvigenin, and spathuleno)	–	*In vivo* and *in vitro*	Methanolic extract *L. caulescens* (MELc) at 38 and 120 mg/kg induced a significant decrease in heart rate, SBP, and DBP in comparison with control, captopril (30 mg/kg). MELc (120 mg/Kg) induced a long-term antihypertensive and vasorelaxant effect.	(Estrada-Soto et al., 2012)
***L. aspera* (Willd.) Link**	Leaf	Ethanolic extract of leaf	Phytosterols and alkaloids	*In vivo*	The ethanolic extract of leaves (200 and 400 mg/kg) showed significant inhibition against dexamethasone-induced hyperlipidemia in rats by maintaining the serum levels of cholesterol and triglycerides near to normal levels.	(Kumar, 2016)
***M. officinalis* L**.	Leaves	Hydroxycinnamic acid derivatives (rosmarinic acid **(5)** and caffeic acids **(6)**)	Phenol	*In vitro*	The aqueous extract (1–1,000 µg/mL) exhibited concentration-dependent relaxation in phenylephrine-precontracted endothelium intact thoracic rings. Rosmarinic acid **(5)** possessed a dose-dependent vasorelaxant effect.	(Ersoy et al., 2008)
***M. macrosiphon* Coss. (syn. *S. macrosiphon* (Coss.) Maire)**	Aerial parts	Methanolic extract (carvacrol, thymol, flavonoids, beta-caryophyllene, gamma-terpinene, and linalool)	Isopropanoids	*In vitro*	The methanolic extract of its flowering stage possessed higher activity in inhibiting α-carotene oxidation with an EC_50_ value of 57.0 ± 0.6 mg/mL.	(Amiri, 2011)
***O. vulgare* L**.	Whole plants	Flavonoids and phenolic acids, rosmarinic acid **(5)**, origanoside	Phenolic compounds	*In vitro*	The *in vitro* test of the phenolic compounds exhibited potent DPPH radical scavenging activities with SC_50_ values ranging from 16.7 ± 1.1 to 221.8 ± 49.0 µM.	(Zhang et al., 2014)
***Orthosiphon aristatus* (Blume) Miq. (syn. *Orthosiphon stamineus* Benth.)**	Leaves	Methylripariochromene (MRC)	–	*In vivo*	MRC (50 and 100 mg/kg) reduced SBP and heart rate of the mice *via* the subcutaneous route. The MRC reduced BP due to the dilation of the blood vessel decreased in cardiac output. It also has suppressive actions to contractions.	(Matsubara et al., 1999)
	Whole plant	Sinensetin **(17)**, eupatorin **(26)**, flavonoid	–	*In vitro*	Sinensetin **(17)** (0.03–2.11 µM) caused concentration-dependent vasorelaxation of phenylephrine-contracted endothelium-intact aortic rings. *O. aristatus* caused aortic ring that was pre-contracted with phenylephrine in the presence and absence of endothelium to be relaxed. It also caused relaxation to the aortic ring that was pre-contracted with potassium chloride in the endothelium-intact aortic ring.	(Yam et al., 2016; Yam et al., 2018)
***Phlomoides bracteosa* (Royle ex Benth.) Kamelin and Makhm. (syn. *Phlomis bracteosa* (Royle ex Benth.) Kamelin and Makhm.)**	Whole plant	Marrubiin **(20)** (labdane-type diterpene) and phlomeoic acid (tricyclic clerodane-type diterpenoid)	–	*In vitro*	It exhibited a vasodilator effect mediated through dual Ca^2+^ channel inhibition (endothelium-independent) and nitric oxide (NO) generation (endothelium-dependent) pathways. Marrubiin **(20)**, phlomeoic acid, RA, and RB inhibited the K^+^ and PE-induced contractions in endothelium-denuded rings with different patterns.	(Khan et al., 2012)
***Plectranthus hadiensis* (Forssk.) Schweinf. ex Sprenger (syn. *Coleus forskohlii* Willd.)**	Whole plant	Ethanolic extract	Diterpene	*In vivo*	Coleonol produced well-marked and sustained hypotension in the anesthetized cat in a dose range of 0.1–1.0 mg/kg, given intravenously in the smooth muscle. A close intra-arterial injection of coleonol (0.1 mg) increased the blood flow in the femoral artery.	(Dubey et al., 1981)
***P. monostachyus* (P. Beauv.) B. J. Pollard (syn. *S. monostachyus* (P. Beauv.) Briq.)**	Leaves	Ethanolic extract	flavonoids, coumarin, polyphenol	*In vivo*	The extract (0.6–17.6 mg/kg bw) induced a significant decrease in arterial blood pressure (EC_50_ = 2.5 ± 0.15 mg/kg b.w) in a dose-dependent manner (p < 0.001). The extract (10^−2^-1 mg/mL) inhibited aorta smooth muscle contraction suggesting calcium channel blocking action with a major inhibitory effect on L-type voltage-operated Ca^2+^ channels.	(Fidele et al., 2012)
***Pogostemon elsholtzioides* Benth**.	Leaves	Essential oil (Curzerene, majority sesquiterpenes)	–	*In vitro*	The essential oil of *P. elsholtzioides* induced dose-dependent vasodilation in pre-contracted aortic rings against contraction evoked by Phe (10^−3^M). Injection of the essential oil at the dose level 20 mg/kg induced a significant decrease in MAP and heart rate.	(Shiva Kumar et al., 2017)
***P. vulgaris* L**.	Whole plant	Rosemarinic acid	–	*In vivo* and *in vitro*	The aqueous extract showed a substantial increase in the HDL level. The extract exhibited radical scavenging activity towards superoxide, hydroxyl, and hydrogen peroxide.	(Zargar et al., 2017)
***R. officinalis* L**.	Leaves	*n*-butanol extract	Flavonoids and phenolic	*In vivo*	The *n*-butanol extract (400 mg/kg) significantly reduced (p < 0.01) the plasma total cholesterol level of diabetic mice group by 51.85% reduction.	(Belmouhoub et al., 2017)
***S. miltiorrhiza* Bunge**	Root	Salvianolic acid **(23)**	Phenolic acids	*In vivo* *In vitro*	Salvianolic acid **(23)** reduced thrombus weight and increased plasma CAMP level. Salvianolic acid **(23)** (2.5–10 mg/kg) administered inhibited the platelet aggregation in a dose-dependent manner. The acid inhibited various agonists that stimulate platelet aggregation. It also induced CAMP levels in platelets that were activated by ADP. induce a rise in CAMP level in platelets activated by ADP.	(Fan et al., 2010)
***S. ofﬁcinalis* L**.	Shoots	Phenolic acids, carnosol derivatives, and flavonoids, namely, rosmarinic acid **(5)**, carnosic acid, and carnosol **(10)** followed by caffeic acid **(6)**, rosmanol **(9)**, rosmadial, genkwanin, and cirsimaritin	Phenolic compound and flavonoids	*In vitro*	It exhibited scavenging activity towards active oxygen’s such as superoxide anion radicals, hydroxyl radicals, and singlet oxygen, and inhibits lipid peroxidation.	(Masaki et al., 1995; Santos-Gomes et al., 2002)
***Salvia scutellarioides* Kunth**	Leaves and stem	Aqueous plant extract	–	*In vivo*	Intravenous consumption of 1 and 2 g/kg of the extract produced a significant increase in diuresis. It increased the urinary excretion of potassium and chloride. High tubular concentrations of potassium stimulated the activity of the Na^+^/K^+^ ATPase pump in the basolateral membrane of the tubular epithelial cells, decreasing the sodium concentration in the urine.	(Ramirez et al., 2006)
***Satureja cuneifolia* Ten. (syn. *Satureja obovata* Lag.)**	Whole plant	Eriodictyol **(21)**	Flavonoid	*In vitro*	Eriodictyol **(21)** inhibited the KCl and noradrenaline-induced contraction in a concentration-dependent manner. Eriodictyol **(21)** (10^5^, 5 ×10^5^, 10^4^, and 5 × 10^4^ M) added before the contraction reduced the tonic phase of contraction.	(De Rojas et al., 1999)
***Sideritis raeseri* Boiss. and Heldr**.	Aerial parts	Plant extract	Terpenoids, sterols, coumarins, flavonoid aglycones, and glycosides	*In vivo* *In vitro*	The extract (0.025–7.5 mg/kg) caused a dose dependent decrease of the arterial pressure and heart rate, with an EC_50_ value of 24.31 ± 3.87 mg/kg and 88.14 ± 7.51 mg/kg, respectively Extract of *S. raeseri* (0.005–1.5 mg/mL) elicited a vasodilator action (EC_50_: 0.11 ± 0.008 mg/mL).	(Kitic et al., 2012)
***T. polium* L**.	Aerial parts	Aqueous extract	Diterpenoids, flavonoids, iridoids, sterols, and terpenoids	*In vivo*	Administration of 50 to 150 mg/kg of the extract for ten days significantly reduced the serum levels of cholesterol and triglycerides in hyperlipidemic rats dose-dependently.	(Rasekh et al., 2001)
***Thymus saturejoides* Coss**.	Whole plant	Caffeic acid **(6)** Rosmarinic acid **(5)** Quercetin **(3)**	Polyphenolic compound	*In vivo* and *in vitro*	The extract exhibited a hypolipidemic effect. Injection of 0.2 g/100 g of the extract significantly lowered both plasma triglycerides and cholesterol levels after 24 h of treatment. The reduction of plasma total cholesterol was associated with a decrease in the LDL fraction. It suppressed the elevated blood concentrations of triglycerides.	(Ramchoun et al., 2012; Khouya et al., 2015)
***Thymus serpyllum* L**.	Whole plant	GAE, and rosmarinic and caffeic acids **(6)**	Phenols and flavonoids	*In vivo* *In vitro*	The injection *via* bolus of 100 mg/kg body weight produced a significant decrease in SBP, DBP, and total peripheral resistance. Rosmarinic acid **(5)** portrayed a dose-dependent antioxidant activity against *in vitro* LDL oxidation. It inhibited the formation of conjugated dienes and TBARS. The thyme extract (1 mg/mL) exhibited nitric oxide (NO) scavenging activity of 63.43%, with the IC_50_ value of 122.36 µg/mL.	(Katalinic et al., 2006; Mihailovic-Stanojevic et al., 2013)
***Thymus zygis* L**.	Whole plant	Caffeic acid **(6)** and rosmarinic acid **(5)**	–	*In vivo*	In the aPTT test, it completely inhibited the plasma clot formation in the concentration of 5.72 mg/mL in the clotting mixtures and prolongs the clotting time at the concentration of 0.18 mg/mL In the PT test, it completely inhibited the clotting process at a concentration of 11.43 mg/mL.	(Khouya et al., 2015)
***Vitex megapotamica* (Spreng.) Moldenke**	Leaves	Crude extract	Flavonoid	*In vivo*	The hydroethanolic extract (500 or 1,000 mg/kg/day) significantly reduced the levels of total cholesterol, triglycerides, LDL-C, and the atherogenic index. The atherosclerotic plaque formation was impaired only by the lower dose of the hydroethanolic extract.	(Pires et al., 2018)
***Ziziphora clinopodioides* Lam**.	Whole plant	Caffeic acid **(6)**, luteolin **(8)**, 7-methylsudachitin, thymonin	Phenolic and flavonoid	*In vitro*	The extract exhibited relaxation on the vascular smooth muscle cells through intracellular and extracellular Ca^2+^ mobilization. It acts on voltage-dependent K^+^channels.	(Senejoux et al., 2010)

## Inhibitory Activity on Cardiovascular Diseases

### Antioxidant Activity

The chemical substances that reduce or prevent oxidation are known as antioxidants. Antioxidants can resist the free radicals from causing damaging effects in tissues. They are often used to safeguard cerebrovascular diseases (Bandyopadhyay et al., 2007). The oxidative stress that occurs at the cellular level acts as the prime pathogenic factor for cardiovascular diseases. It occurs due to the free radical’s toxic being released by the vascular smooth muscle cells and endothelial cells (Fearon and Faux, 2009). Apart from that, cardiovascular diseases are caused by oxidative stress by reactive oxygen species (ROS) such as uncoupled nitric oxide synthases, xanthine oxidase, and NADPH oxidases (Taleb et al., 2018). ROS voluntarily attack and cause oxidative damage to various biomolecules such as lipoproteins, lipids, protein, and DNA.

This oxidative harm is an essential etiological figure that ensnared a few endless human sicknesses. Examples are cardiovascular illnesses, rheumatism, diabetes mellitus, cerebrovascular infections, and malignant growth. The damage of DNA and oxidative stress may be caused by oxidized low-density lipoproteins (oxLDL) or by hypercholesterolemia due to individual diet lifestyle (Ceaser et al., 2003). One of the lead causes of atherosclerosis is oxygen-free radicals that reduce the cells’ capacity to inhibit the oxidation. This can leads to fatal inflammation disorder.

Most of the plants from the Asteraceae and Lamiaceae plant family were proven to possess the antioxidant effect *via in vitro* assay such as 2,2-diphenyl-1-picrylhydrazyl (DPPH) assay. The plants from the Asteraceae family that shows antioxidant effect based on this study are *Achillea tenuifolia* Lam. (syn. *Achillea santolina* L.)*, Anthemis melampodina* subsp. *deserti* (Boiss.) Eig (syn. *Anthemis deserti* Boiss)*, Artemisia absinthium* L.*, Baccharis trimera* (Less.) DC, *C. crepidioides* (Benth.) S. Moore, *Helichrysum leucocephalum* Ausfeld*, Laggera decurrens* (Vahl) Hepper and J. R. I. Wood*, Senecio ovatus* subsp. *stabianus* (Lacaita) Greuter (syn. *Senecio stabianus* Lacaita), and *Silybum marianum* (L.) Gaertn. The plants from Lamiaceae that show antioxidant activities are *Ajuga iva* (L.) Schreb.*, Ballota glandulosissima* Hub. -Mor. and Patzak*, Dracocephalum moldavica* L.*, H. leucocephalum* Ausfeld, *Lavandula angustifolia* Mill., *Lavandula stoechas* L.*, Micromeria macrosiphon* Coss. (syn. *Satureja macrosiphon* (Coss.) Maire), *Origanum vulgare* L.*, Plectranthus monostachyus* (P. Beauv.) B. J. Pollard (syn. *Solenostemon monostachyus* (P. Beauv.) Briq), and *Salvia ofﬁcinalis* L. These plants had shown the antioxidant effect, with its scientific value. Based on the studies, antioxidant properties were mostly exhibited by phenolic compounds due to their tendency to scavenge the free-radicals. The phenolic compounds act by chelating the metal ions, improving the endogenous antioxidant system, and avoiding the formation of free radicals. Due to this, studies on the search for natural antioxidants from plant origin have become more intense in recent years (Seyoum et al., 2006). Other chemical compounds in these plants that mainly contribute to its antioxidant activity are flavonoids, flavanols, and diterpenes.

*Achillea tenuifolia* Lam. is known for its free radical scavenging activity. The ferric thiocyanate test was conducted to determine the antioxidant activity of *A. tenuifolia* extract (ATE) by measuring peroxide concentration during the early stage of lipid peroxidation (Ardestani and Yazdanparast, 2007). ATE concentration of 200 and 400 µg/mL suppress the lipid peroxidation by the extension on the lag phase and the reduction of the propagation rate, which reflects the typical chain-breaking antioxidant characteristics. The activity was determined using a linoleic acid system. The peroxidation activity was measured using the thiocyanate method by using the absorbance at 500 nm to determine the peroxide values. The study showed that the ATE contained 55 mg ascorbic acid equivalents per gram of extract with the EC_50_ value of 55 µg/mL (Ardestani and Yazdanparast, 2007). Besides consists of gallic acid **(1)** and catechin **(2)**, this plant also contains phenols and flavonoid, which induce the antioxidant activity in nature (Khafagy et al., 1976).

The methanolic extract of *A. melampodina* subsp. *deserti* (Boiss.) Eig in the concentration of 25, 50, and 100 µg/mL showed more significant DPPH radical scavenging activity, compared to the positive controls, l-ascorbic acid, and butylated hydroxytoluene, BHT (Shahat et al., 2014). The DPPH radical scavenging activity and chelating effect of the plant were concentration-dependent. The plant extract at the concentration of 400 µg/mL showed a 100% chelating effect. The ferrous ion chelating effect of the extract was measured to determine the capacity of the extract to bind to the ferrous ion to catalyze oxidation in lipid peroxidation. The extract also possessed superoxide anion radicals scavenging capacity. It has a potent anion scavenging power at each concentration tested with a range from 25 µg/mL to 400 µg/mL (Shahat et al., 2014).

*Artemisia absinthium* L. methanolic (MAB) extract at the concentration from 25 to 100 µg/mL showed scavenging activity on superoxide anion radicals produced from the PMS-NADH system (Bora and Sharma, 2011). When the MAB was administered orally at doses of 100 or 200 mg/kg, it restored superoxide dismutase (SOD) and glutathione (GSH) levels and decreased the thiobarbituric acid reactive substances (TBARS) level. As a whole, it causes inhibitory activity on oxidative stress induced by cerebral ischemia and reperfusion. GSH is known as a central component in the antioxidant and known as the defense cells. It acts by detoxifying ROS directly and as a substrate for various peroxides (Bora and Sharma, 2011). The plant extract consists of quercetin **(3)**, rutin, and vanillic acids **(4)**. It was reported that the plant manages to alleviate stroke disease by reducing SOD activity in the serum (Spranger et al., 1997). Its antioxidant activity has potential in the acute treatment of cerebral ischemia.

*Ballota glandulosissima* Hub. -Mor. and Patzak is a Turkish Ballota species, collected from Kumluca. The aerial parts of *B. glandulosissima* were extracted using ethanolic extract and were determined its antioxidant activity *via in vitro* (Citoglu et al., 2004). The extract showed remarkable anti-superoxide anion formation by inhibiting the activity with an IC_50_ value of 0.51 mg/mL. In inhibiting lipid peroxidation, the extract exhibited a strong inhibitory capacity and potent scavenging property (IC_50_, 15 mg/mL) compared to α-tocopherol (IC_50_, 3 mg/mL) (Citoglu et al., 2004).

The antioxidant activity of methanolic extract of aerial part of *C. crepidioides* (Benth.) S. Moore was determined by measuring its total phenol and flavonoid content, reducing capacity and radical scavenging activity on DPPH assay (Bahar et al., 2016). The plant consisted of various phytochemicals such as flavonoids, alkaloids, tannins, saponins, glycosides, and reducing sugar which, contributed to the extract’s antioxidant activity. The activity reported was lower (IC_50_: 130.32 µg/mL) compared to ascorbic acid (IC_50_: 11.24 µg/mL). The aerial part extract of the plant consists of gallic acid **(1)** and quercetin **(3)**, which tends to cause the antioxidant effect (Bahar et al., 2016).

*Dracocephalum moldavica* L. contains antioxidant compounds with cardioprotective effects (Jiang et al., 2014). The total ﬂavonoid extract (5 µg/mL) from *D. moldavica* pre-treatment had caused improvement in the heart rate and coronary ﬂow by decreasing lactate dehydrogenase levels, creatinine kinase levels, and caused a rise on left ventricular developed pressure. In the concentration of higher than 70 mg/L, the total flavonoid extract exhibited a higher DPPH radical scavenging activity than vitamin E. The study showed that *D. moldavica* exhibited protection against myocardial ischemia/reperfusion(I/R)-induced injury by enhancing GSH/GSSG ratio and SOD activity and attenuating malondialdehyde (MDA) production (Jiang et al., 2014). The ethanolic extract of *H. leucocephalum* Ausfeld aerial parts showed an antioxidant effect *via in vitro* assay. The extract exhibited antioxidant activity with an IC_50_ value of 69.94 ± 0.17 μg/mL. The high amount of phenolic compounds from this plant showed a sufficient antioxidant activity in the DPPH assay (Goldansaz et al., 2018).

*Laggera decurrens* (Vahl) Hepper and J. R. I. Wood. contains 46.3% of oxygenated monoterpenes and among them are thymol (5.7%) and 3-methoxy-2-methyl-5-(1-methylethyl)-2,5-cyclohexadiene-1,4-dione (3-methoxythymoquinone) (28.1%) (Mothana et al., 2011). The oil consisted of 22.7% oxygenated sesquiterpenes with caryophyllene oxide (3.4%), T-cadinol (5.1%) and eudesma-11-en-4a-ol (7.0%) as the main compounds. The essential oil, especially, at a concentration of 500 µg/mL, exhibited strong antioxidant activity by causing a reduction in DPPH concentration which is comparable to ascorbic acid activity due to the presence of carvacrol, 3-methoxymoquinone, and thymol (Mothana et al., 2011).

*Lavandula angustifolia* Mill. from the Lamiaceae family consists of essential oil that can inhibit isoproterenol-induced myocardial infarction in rats (Ezzoubi et al., 2014). It protected the myocardium against isoproterenol-induced myocardial infarction at the concentration of 20 mg/kg of the essential oil. This amount of oil had caused the reduction in the ST-elevation of the myocardial infarction situation, resembled its effectiveness in cardiovascular disease, and indicated its protective effects on cell membrane functions (Ezzoubi et al., 2014).

The essential oils and subfractions of *M. macrosiphon* Coss. methanolic extract were examined their antioxidative properties *via in vitro* study (Amiri, 2011). In examining the effect of *M. macrosiphon* on DPPH assay, the polar subfraction of the flowering stage was more potent with an EC_50_ value of 57.0 ± 0.6 mg/mL. In contrast, the essential oil of the flowering stage was more potent (EC_50_: 91.7 ± 1.2 mg/mL) in inhibiting α-carotene oxidation with almost similar inhibitory activity compared to the positive control, BHT. The oxidation of α-carotene was determined by observing its discoloration caused by coupled oxidation of linoleic acid and α-carotene that generates free radicals.

The phenolic compounds isolated from the ethanolic extract of the whole plant of *O. vulgare* L. were determined their antioxidant activity. From the study, *O. vulgare* consists of phenol compounds such as 2,5-dihydroxybenzoic acid, 3,4-dihydrobenzoic acid, rosmarinic acid **(5)**, origanoside, maltol, *E*-caffeic acid **(6)**, apigenin **(7)**, luteolin **(8)**, and didymin (Zhang et al., 2014). Based on the *in vitro* test, the phenolic compounds of *O. vulgare* showed potent antioxidant activity. The SC_50_ values of the compounds ranged from 16.7 ± 1.1 to 221.8 ± 49.0 µM. The scavenging activity of the compound that consists of 3,4-dihydroxyphenyl and gastrodin moiety was higher than its positive control, the ascorbic acid. The highest radical scavenging activities were exhibited by compounds with danshensu moieties with SC_50_ values 17.5 ± 1.1 µM and 16.7 ± 1.1 µM. The phenolic structure is responsible for the scavenging activity by donating the hydroxyl group to the free radicals (Graf, 1992). The ferric reduced antioxidant power (FRAP) assay also showed similar antioxidant activity of the phenolic compounds. The FRAP values of the compounds ranged from 143.0 ± 4.0 and 201.0 ± 8.0, with the highest activity exhibited by origanoside and apigenin **(7)** (Zhang et al., 2014).

*Salvia ofﬁcinalis* L., which is also known as sage, contains phenolic compounds in its shoot part (Santos-Gomes et al., 2002). The antioxidant extract of the shoots contains phenolic acids such as gallic acid **(1)**, rosmarinic acid **(5)**, caffeic acid **(6)**, 5-*O*-caffeoylquinic acid, and 3-*O*- caffeoylquinic acid. The identified flavonoids in the extract are hesperetin, genkwanin, hispidulin, cirsimaritin, and apigenin **(7)**. Whereas, the phenolic diterpenes are methyl carnosate, rosmanol **(9)**, epirosmanol, epiisorosmanol ethyl ether, epirosmanol methyl ether, carnosic acid, rosmadial, and carnosol**(10)**. The rosmarinic acid **(5)**, carnosic acid, and carnosol **(10)** contributed to *S. ofﬁcinalis* inhibitory activity on lipid peroxidation (Santos-Gomes et al., 2002). The ethanolic leaves extract of the plant contains a high amount of chlorogenic acid **(11)** and rosmarinic acid **(5)**. The compounds exhibited strong antioxidant activity due to the ability of their phenols structure to donate hydrogen atoms to free radicals. (Ramu et al., 2012).

*Senecio ovatus* subsp. *stabianus* (Lacaita) Greuter was used an *in vitro* assay to examine its antioxidant activity by conducting azino-bis(3-ethylbenzthiazoline-6-sulphonate) (ABTS) and DPPH assay (Tundis et al., 2012). The study showed that the ethyl acetate extract of the plant exhibited the strongest activity on ABTS (IC_50_ value: 32.7 mg/mL) and DPPH (IC_50_ value: 35.5 mg/mL) assay. Based on the Folin-Ciocalteu method, the plant contains total phenol of 76.3 mg chlorogenic acid **(11)** equivalent per gram of the plant. Whereas, based on the formation of the flavonoid-aluminum complex method, the plant contains a total flavonoid of 11.8 mg quercetin **(3)** equivalent per gram of the plant. Thus, it was assumed that the antioxidant activity of the plant due to its flavonoids content (Tundis et al., 2012).

*Silybum marianum* (L.) Gaertn. contains silymarin **(12)**, which is one of the polyphenolic antioxidants. Administration of 200 mg/kg silymarin **(12)** by intraperitoneal on rats reduced ROS level and protected the rats from supra celiac abdominal aorta ischemia or reperfusion injury. (Kocarslan et al., 2016). Administration of 100 mg/kg silymarin **(12)** on the rats reduced iron and oxidative stress level of the rats’ blood. Besides, the phenolic structure of silymarin **(12)** caused the compound to has a strong scavenger activity towards hypochlorous acid (HOCI). It inhibited hydroxyl radical formation, which is essential for the inhibition of xanthine oxidase activity (Varga et al., 2006). A low concentration of silymarin **(12)** caused inhibition of the NF-kB pathway by treating and attenuating the inﬂammatory reaction that stimulates atherosclerosis.

### Antihyperlipidemia, Hypolipidemia, and Hypocholesterolemia Activity

The rise in both blood cholesterol and triglyceride that may be due to hereditary factors is known as hyperlipidemia. One of the CVD that is caused by hyperlipidemia is atherosclerosis. It is the condition where the lipids or fat substances which are denoted as plagues hardened the arteries. These plagues will be built up in the walls of arteries and lead to the narrowing of the arteries. It will diminish the ability of blood flow in the artery that usually associated with vascular diseases, heart disease, and stroke (Akinpelu et al., 2016). The role of substance that possesses the antihyperlipidemic effect is to reduce the total cholesterol level in the body by reducing triglycerides, very low-density lipoprotein (VLDL) and low-density lipoprotein (LDL). The antihyperlipidemic possessing substance also has a role in increasing the high-density lipoprotein (HDL) level in the body, which is known as the good cholesterol in the body that alleviates the risk of CVD.

A few plants from the plant family of Asteraceae and Lamiaceae plant family possess an antihyperlipidemic effect. Of such, the plants from Asteraceae *are Achillea arabica* Kotschy (syn. *Achillea biebersteinii* Hub.-Mor.)*, Ageratum conyzoide* L.*, Chromolaena odorata* (L.) R. M. King and H.Rob.*, C. crepidioides* (Benth). S. Moore*, C. cardunculus* L. (syn. *Cynara scolymus* L.)*, Eclipta prostrata* (L.) L.*, E. praetermissa* Milne-Redh*, Gundelia tournefortti* L*., Gymnanthemum amygdalinum* (Delile) Sch. Bip. (syn. *Vernonia amygdalina* Delile), *Inula racemosa* Hook F.*, Launaea intybacea* (Jacq.) Beauverd (syn. *Lactuca runcinata* DC.)*, Solidago chilensis* Meyen*, Sphaeranthus indicus* L., and *Vernonia elaeagnifolia* DC. The plants from the Lamiaceae family are *Clerodendrum volubile* P. Beauv.*, Lagenaria siceraria* (Mol.) Standl.*, Lallemantia royleana* Benth.*, Leucas aspera* (Willd.) Link, *Prunella vulgaris* L.*, Rosmarinus officinalis* L.*, Teucrium polium* L.*, Thymus dreatensis* Batt. (syn. *Thymus atlanticus* (Ball) Pau), and *Vitex megapotamica* (Spreng.) Moldenke. These plants’ extracts from different solvents, such as ethanol, methanol, and water lower the lipid markers concentration in the body either *via in vivo* or *in vitro* assays.

*Achillea arabica* Kotschy ethanolic extract was used to test its hypolipidemic effect in animals (Mais et al., 2016). The extract was from the aerial parts of the plant taken during its flowering phase. The high-fat diet was fed to adult male Golden-Syrian hamsters for ten days to cause hyperlipidemia. The dose of 400 mg/kg of the *A. arabica* ethanolic extract had reduced VLDL, cholesterol, LDL, and triglycerides level in the hamsters’ serum. It had no significant effect on HDL. Total cholesterol and triglycerides in the hepatic were also reduced. The plant extract contains flavonoids, sesquiterpene lactones, and polyphenols. Whereas, its essential oil contains a high amount of eucalyptol **(13)** (10.98%), camphor **(14)** (12.46%) and piperitone **(15)** (31.06%). These may act as an inducer for the hypolipidemic effect of *A. arabica* upon the experimental hyperlipidemic hamster (Mais et al., 2016). Few reports had documented that flavonoids and phenolic compounds have antioxidant, antihyperlipidemic, and antihypertensive activity as their pharmacological effect (Rouhi-Boroujeni et al., 2015).

*In vivo* study of methanolic extracts of *Ageratum conyzoide* L. root, leaf, and stem were carried out on rats to examine its hypolipidemic activity (Atawodi et al., 2017). The extracts contain flavonoids, alkaloids, cardiac glycosides, triterpenes, saponins, carbohydrates, and tannins. Meanwhile, the leaf also consists of steroids. Fiber, saponins, and flavonoids have an underlying antihyperlipidemic effect. The methanolic extract in a concentration of 100 mg/kg was treated on rats. The extract reduced serum lipids, which is one of the insulin-releasing factors. Insulin inhibits lipolysis, thus causing a rise in uptake of fatty acids into adipose tissue and triglyceride synthesis. The diabetic rat had shown a significant reduction in total cholesterol, triglycerides, low-density lipoprotein cholesterol (LDL-C) levels, and increased high-density lipoprotein cholesterol (HDL-C) (cardioprotective lipid) levels (Atawodi et al., 2017).

*Chromolaena odorata* (L.) R. M. King and H.Rob. leaves aqueous extract was examined its atherogenic indices and plasma lipid profiles on rats that have fed loaded with cholesterol (Ikewuchi and Ikewuchi, 2011). The intra-gastric gavages of the plant extract (100 mg/kg body weight) was administered to the rats. As a result, there was a significant reduction in total cholesterol, LDL, non-HDL, VLDL, and plasma’s triglyceride levels. The HDL-C level in the plasma was high once being treated with the extract. The extract contains saponin, which was reported to have a hypercholesterolemic activity (Soetan, 2008). The extract significantly reduced (p < 0.05) the atherogenic index of plasma, cardiac risk ration, and atherogenic coefficient compared to the control group. Thus, the study showed that *C. odorata* could reduce the risk of heart diseases (Ikewuchi and Ikewuchi, 2011).

Ethanolic leaf extract of *C. volubile* P. Beauv. was evaluated by its anti-hyperlipidemic activity (Akinpelu et al., 2016). *Clerodendrum volubile* ethanolic leaf extract contains cardiac glycosides, saponins, flavonoids, alkaloids, tannins, and steroids. The extract contains phenolic compounds as the major constituents, followed by flavonoids and alkaloids. The lowest constituent of the extract was tannin, followed by saponin. The extract with a concentration of 250 and 500 mg/kg was administered to the hyperlipidemic animal. The extract significantly reduced the triglycerides, VLDL, total cholesterol, and LDL level in curative and phyto-preventive animals in addition to increased their HDL level (Akinpelu et al., 2016).

The methanolic extract of the aerial parts of *C. crepidioides* (Benth.) S. Moore had shown an antihyperlipidemic activity (Bahar et al., 2016). The albino rats were induced with a high-fat diet to mimic hyperlipidemia model. The methanolic extract in a concentration of 150 and 300 mg/kg were administered orally to the rats. As a result, the extract increased HDL-C level while reduced VLDL-C, LDL-C, total cholesterol, and triglycerides levels compared to the positive group. The extract in a concentration of 300 mg/kg showed potent antihyperlipidemic activity compared to the positive control group (Bahar et al., 2016).

*Cynara cardunculus* L. is known for its antiatherogenic and hypolipidemic effects. Hypercholesterolemic rats were fed with *C. cardunculus* leaves aqueous extract at the amount of 150, 300, and 60 mg/kg (Mocelin et al., 2016). The extracts exhibited DPPH scavenging activity with an IC_50_ value of 57.40 ± 2.05 µg/mL. After four weeks of treatment, the serum lipid profile showed that there was a decrease in total cholesterol and LDL-C levels. The flavonoids and phenols content decreased the activities of acyl-CoA acetyltransferase and HMG-CoA reductase in the hypercholesterolemic rats. It decreased the availability of cholesterol esters to form VLDL, which caused a reduction in the secretion of VLDL from the liver (Mocelin et al., 2016).

*Eclipta prostrata* (L.) L. leaf extract was measured its hypolipidemic activity (Dhandapani, 2007). The phytochemical screening had shown the presence of saponins, alkaloids, flavonoids, phytosterols, and tannins in the leaf extract. Atherogenic diet caused an increase in total cholesterol and total protein and decreased serum HDL-cholesterol level in rats. Daily administration of the aqueous extract in the dose 100 and 200 mg/kg increased the rats’ HDL level and reduced protein level, triglycerides, and total cholesterol significantly. It had also shown an increase in the atherogenic index. The saponins may contribute to the hypolipidemic effect of the plant extract (Dhandapani, 2007).

*Emilia praetermissa* Milne-Redh had shown its antihyperlipidemic effect *via* its aqueous leaf extract. The extract at doses of 100 mg/kg, 200 mg/kg, and 400 mg/kg were fed to male albino rats. As a result, the extract reduced the levels of plasma atherogenic index, total cholesterol, LDL, and triglycerides, and raised the level of HDL significantly when compared with the hyperlipidemic group. The hypolipidemic activity might due to the cholesterol-lowering effect of the extract, which may be portrayed by tannins, terpenoids, and flavonoids content. Terpenoid acts as an intermediate in cholesterol synthesis. It regulates the degradation of HMG-CoA reductase activity, which is the main enzyme in cholesterol synthesis (Ngozi et al., 2013).

Alcohol extract (IrA) and hexane extract (IrH) of *Inula racemosa* Hook F. roots at the dose of 100 mg/kg was administered to guinea pigs to observe the extracts’ hypolipidemic effect (Mangathayaru et al., 2009). IrA showed antihyperlipidemic activity by reducing lipid peroxidation and lipid uptake. It decreased foam cell formation and led to a reduction of fatty streak formation. Meanwhile, IrH exhibited more significant activity in enhancing HDL-C (p < 0.001) than in reducing LDL levels. Based on the effects of the extracts on coronary artery histopathology, IrA and IrH had shown a replacement in a muscular pattern which is the type of primary medial destruction in early atherosclerosis. It also caused the cardiac tissue to be devoid of fatty degeneration. The presence of the phenolic compounds contributes to the inhibition of LDL oxidation and prevent the degradation and uptake of oxidized LDL by macrophages (Mangathayaru et al., 2009).

*Launaea intybacea* (Jacq.) Beauverd exhibited curative and preventive activity against hyperlipidemia (Devi and Muthu, 2015). Its ethanolic extract of the whole plant reduced cardiac risk ration, plasma lipid, and lipoprotein profile. It increased the HDL level, which responsible for neutralizing atherogenic effects of oxidized LDL, in addition to inhibiting LDL oxidation and reversing cholesterol transport. It also reduced free cholesterol and ester levels. Its lipid-lowering activity was due to its inhibition of hepatic cholesterogenesis or due to its ability to increase fecal sterol excretion (Devi and Muthu, 2015).

### Vasorelaxant and Vasodilation Action

Excessive contraction of vessels can cause increases in pressure that may lead to hypertension. Vasorelaxant facilitates the vasodilation of the contracted vessel to ensure the ease of blood flow through the blood vessels. Vascular smooth muscle relaxation is one of the mechanisms for treatment and prevention of hypertension, with most of the treatments were focusing on impeding vascular smooth muscle contraction (Goodman, 1996). A few plant species from Asteraceae and Lamiaceae exhibited vasorelaxant activity. The plants from the Asteraceae family are *Artemisia campestris* L.*, Bidens pilosa* L.*, Chrysanthemum* x *morifolium* Ramat. Hemsl., and *Pectis brevipedunculata* Sch. Bip. The plants from the Lamiaceae family are *Agastache mexicana* (Kunth.) Lint. and Epling*, C. vulgare* L. (syn. *Calamintha vulgaris* (L.) Druce)*, Isodon rugosus* (Wall. Ex Benth.) Codd*, Lepechinia caulescens* (Ortega) Epling*, Melissa officinalis* L.*, Orthosiphon aristatus* (Blume) Miq. (syn. *Orthosiphon stamineus* Benth.)*, Phlomoides bracteosa* (Royle ex Benth.) Kamelin and Makhm. (syn. *Phlomis bracteosa* (Royle ex Benth.) Kamelin and Makhm.)*, Plectranthus hadiensis (*Forssk.) Schweinf. ex Sprenger (syn. *Coleus forskohlii* Willd.)*, Pogostemon elsholtzioides* Benth*, Satureja cuneifolia* Ten. (syn. *Satureja obovata* Lag.)*, Sideritis raeseri* Boiss. and Heldr., and *Ziziphora clinopodioides* Lam.

*Agastache mexicana* (Kunth.) Lint. and Epling is a medicinal plant species from the Lamiaceae family that can treat hypertension and anxiety conditions. The antihypertensive activity of the dichloromethane extract of *A. mexicana* (DEAm) and its isolated compound, ursolic acid were determined in the male rat (Flores-Flores et al., 2016). The extract exhibited relaxant activity on noradrenaline bitartrate 0.1 µM and potassium chloride (KCl) 80 mM pre-contracted aortic rings which suggest that the extract exhibited vasodilation effect through several receptors, such as the augment of free cytosolic Ca^2+^levels. The extract inhibited the vasoconstriction caused by noradrenaline bitartrate and potassium chloride (Hernandez-Abreu et al., 2013). Ursolic acid evoked a significant decrease in systolic blood pressure (SBP) and diastolic blood pressure (DBP) with no change in the hypertensive rat. This action was due to its diuretic effect in relieving the hypertension condition (Somova et al., 2003).

The aqueous extract of *Artemisia campestris* L. aerial parts (AcAE) has a hypotensive and antihypertensive effect due to its vasodilatory effect (Dib et al., 2017). The extract contains a high amount of polyphenols such as mono-and di-cinnamoyl compounds with the highest concentration of 3,5-dicaffeoylquinic (isochlorogenic A) as its constituent. Meanwhile, the major flavonoids in the extract were (5-caffeoylquinic) chlorogenic acid **(11)** and vicenin-2 (apigenin 6,8-di-C-glucoside) **(16)**. Daily administration of 150 mg/kg of AcAE on *L*-NAME hypertensive rats prevented hypertension by reducing SBP from 170 to 114 mm Hg. The extract at the dose of 40 mg/kg reduced SBP and DBP without affecting heart rate. The extract caused vasorelaxation *via* inhibition of calcium influx through voltage-operated calcium channels and the calmodulin-NO-sGC-PKG pathway. Besides, the extract also activated intracellular calcium mobilization into the sarcoplasmic reticulum (Dib et al., 2017).

Based on one of the studies on *Bidens pilosa* L., the plant exhibited a vasorelaxant effect on precontracted rat aorta induced by the KCl (Nguelefack et al., 2005). Besides, it also exhibited vasodilating activity on norepinephrine-induced tonic contraction. The endothelium of the vascular managed to secrete contractile factors and relaxant that caused regulation of vascular tone. The chemical and physical stimulations are responded by the endothelial cells, by producing prostacyclin, nitric oxide, and bradykinin which are the relaxant factors (Corvol et al., 1993; Dimo et al., 2001).

*Clinopodium vulgare* L. was used in *in vivo* and *in vitro* studies to understand its antihypertensive activity (Khan et al., 2018). The administration of *C. vulgaris* crude extract and fractions on normotensive and high salt-induced hypertensive rats reduced the rats’ mean arterial pressure (MAP). It has a distinct effect on hypertensive rats compared to the normotensive rats. At the dose of 1, 3, 10, and 30 mg/kg, the extract had shown an antihypertensive effect in hypertensive rats with the most significant activity exhibited by the extract at the dose of 10 and 30 mg/kg. The vasodilatory effect of the extract (EC_50_:0.27 mg/mL) in the extracted rat aorta was endothelium-dependently. The extracts worked by inhibiting the high K^+^ precontraction and rightward shifted Ca^2+^ concentration-response curves which have an identical mechanism to verapamil. The antihypertensive effect that was showed by *C. vulgaris* is due to the vasodilation effect that involves muscarinic receptor-linked NO and activation of tetraethylammonium (TEA)-sensitive K^+^ channels, Ca^2+^ antagonism, and prostacyclin. The methanolic extract of the plant consists of quercetin **(3)** and rutin, which may act as the substance that possesses the vasodilatory effect (Khan et al., 2018).

Flower extract of *Chrysanthemum* x *morifolium* Ramat. Hemsl. exhibited a vasodilatory effect by reducing the blood pressure of cardiac hypertrophy rats (Gao et al., 2016). The major phytochemicals in this extract are 4,5-di-caffeoylquinic acid, 3,5-dicaffeoylquinic acid, luteolin-7-β-glucoside, 3-chlorogenic acid **(11)**, and apigenin-7-*O*-glucoside. A range of 75 to 150 mg/kg extract was fed to the rats for four weeks to study the effect of the extract on the rats’ SBP. The dose of 150 mg/kg showed a reduction in the SBP, which was about 4% by the second week. One month administration of the extract caused a reduction in the serum-free fatty acid (FFA) by 18.9% to 29.8%, and myocardial FFA level by 5.4% to 16.0%. In addition to the extract activity in inhibiting myocardial hypoxia-inducible factor-1α (HIF-1α) expression, the extract also caused subsequent modulation of some peroxisome proliferator-activated receptor α (PPARα)-mediated gene expression; a decreased in the glucose transporter-4 (GLUT-4) protein expression and an increased in the pyruvate dehydrogenase kinase-4 (PDK-4) and carnitine palmitoyltransferase-1a (CPT-1a) protein expression. (Gao et al., 2016).

*Orthosiphon* aristatus (Blume) Miq. contains sinensetin **(17)**, which is essential for vasorelaxation activity (Yam et al., 2016; Yam et al., 2018). The studies measured the vasorelaxant effect of the compound by conducting a pre-contraction aortic ring assay. The presence of antagonists has shown the mechanism of the vasorelaxant effect of sinensetin **(17)**. Sinensetin **(17)** had exhibited a relaxation effect of potassium chloride-induced endothelium-intact aortic rings and phenylephrine-induced aortic ring with or without the endothelium. The study showed that sinensetin **(17)** exhibited a vasorelaxant effect *via* antagonization of aortic ring contraction through direct and indirect vasorelaxation. The pathways that were involved in this vasorelaxant activity were NO/sGC/cGMP pathways. Sinensetin **(17)** at a dose of 0.262 µg/mL caused the vasodilatory effect (Yam et al., 2016; Yam et al., 2018).

The vasodilatory effect of essential oil of the *Pectis brevipedunculata* Sch. Bip aerial parts (EOPB) was identified (Pereira et al., 2013). The essential oil is rich with citral content, which consists of neral **(18)** and geranial **(19)**, followed by limonene and α-pinene. The vasodilator activity of EOPB and citral was measured using aortic rings obtained from Wistar Kyoto (WKY) rats. EOPB and citral exhibited relaxation to the phenylephrine-induced endothelium-intact aortic rings with an IC_50_ value of 0.044 ± 0.006% and 0.024 ± 0.004%, respectively. Meanwhile, EOPH and citral exhibited relaxation to the phenylephrine-induced denuded aortic rings with an IC_50_ value of 0.093 ± 0.015% and 0.021 ± 0.004%, respectively. The extract mechanism of activity was through the NO/cyclic GMP pathway. Meanwhile, the citral mechanism of activity was by blocking voltage-dependent L-type Ca^2+^ channels that reduced calcium influx. The high concentrations of EOPB caused vasorelaxation of endothelium-independent which predominated the endothelium-dependent pathway (Pereira et al., 2013).

*Phlomoides bracteosa* (Royle ex Benth) Kamelin and Makhm. has few phytochemicals which had the vasorelaxant effects, which are marrubiin **(20)**, phlomeoic acid, and new components (RA and RB) (Khan et al., 2012). The whole plant was powdered and extracted using methanol to produce its extract and examined its activity using rat thoracic aorta. The EC_50_ values of 23.4 and 36.7 μg/mL of marrubiin **(20)** had shown a relaxant effect upon the phenylephrine-induced contraction and inhibited the K^+^. Marrubiin **(20)** in the concentration of 3.0 to 10 μg/mL induced rightward shift of the Ca^2+^ channels. Marrubiin **(20)**, phlomeoic acid, and RA exhibited a more potent effect against K^+^-induced contractions, compared with phenylephrine, which indicated that it had a greater efﬁcacy in blocking the voltage-sensitive Ca^2+^ channels. Among all four phytochemicals studied, the marrubiin **(20)** was most potent for its vasodilator activity. It can be used in further studies to test its extent of vasodilation activity in the future (Khan et al., 2012).

*Satureja cuneifolia* Ten. is a plant species from the Satureja genus. Its constituent, eriodictyol **(21)**, possessed a vasodilatory effect in the rat aorta (De Rojas et al., 1999). The concentration of eriodictyol **(21)** 10^5^ M and 5 × 10^5^ M showed the inhibitory effect of calcium chloride, CaCl_2_ in the concentration-response curve. It possessed a weak inhibition in the calcium from the sarcoplasmic reticulum whereby showing off a light relaxant effect. The final results of the study indicated that the partial mechanism of the vasodilatory effect was due to its inhibition of enzyme protein such as myosin light chain kinase that related to protein kinase C or inhibition of calcium influx (De Rojas et al., 1999).

The dichloromethane extract of *Ziziphora clinopodioides* Lam. (ZDCE) had shown a significant effect on the inhibition of extracellular Ca^2+^ induced contraction in the pre-contracted rings by high KCl and phenylephrine. It also caused an inhibition of intracellular Ca^2+^ release to the phenylephrine. Among the hexane, dichloromethane, and aqueous extracts, ZDCE had shown the endothelium-independent vasodilation properties that occurs due to the extracellular Ca^2+^ influx *via* the voltage and receptor-operated Ca^2+^ channels, causing Ca^2+^ inhibition from the stores of the intracellular and lastly *via* opening the K^+^ channels which are voltage-dependent (Senejoux et al., 2010).

### Anticoagulation and Anti-Thrombosis Activity

A series of zymogens are involved in the blood coagulation process. Proteolysis caused the conversion of zymogens into active enzymes that caused the production of thrombin, which can lead to the conversion of fibrinogen into fibrin (Rand et al., 1996). Enzymes are involved in mediating the blood coagulation of damaged tissues. Factor VII (FVII) binds to uncovered tissue factor (TF), which triggers the development of thrombin that causes coagulation of blood. Anticoagulant inhibits thrombin generation and fibrin formation. An ideal clinical anticoagulant should inhibit thrombin activity without induced bleeding. Platelets and other mediators play an important role in thrombosis and cardiovascular diseases. Based on reported studies, few plants from the plant family of Asteraceae and Lamiaceae plant family possess an anticoagulant effect. The plants’ species from the family of Asteraceae are *Erigeron canadensis* L.*, Flaveria bidentis* (L.) Kuntze*, Leuzea carthamoides* Willd. DC., and *Tridax procumbens* (L.) L. The plants’ species from the Lamiaceae family are *Leonotis leonurus* (L.) *R.Br., S. miltiorrhiza Bunge*, and *Thymus zygis* L.

*Erigeron canadensis* L. consists of different types of flavonoids and tannins on top of the essential oil that is present. Its polyphenolic-polysaccharide preparation was isolated from its flowering part and was determined its anticoagulant activity *via in vivo* assay (Pawlaczyk et al., 2011). The plant preparation had shown its anti-platelet activity specifically towards the cyclooxygenase pathway that was induced by the arachidonic acid (AA), which is similar to acetylsalicylic acid activity. The assay was conducted on standardized human plasma by measuring prothrombin time (PT) and partial thromboplastin time (aPTT). The plant preparation inhibited plasma clot formation in aPTT and PT at the concentration of 390 µg/mL and 1.56 mg/mL, respectively. The plant preparation also exhibited significant anti-IIa activity mediated by the cofactor II of heparin. Further fractionation of the plant preparation at the concentration of 50 µg/mL, showed higher anticoagulation activity in aPTT test corresponded to 7 to 9 IU/mg of 5^th^ International Standard for Unfractionated Heparin (ISUH). *In vivo* studies also showed that the dose of 50 mg/mL of the plant preparation has the anticoagulant effect in the rat. These anticoagulant activities are essential in patients suffering from deep vein thrombosis, and those had already been resistant to the acetylsalicylic derivatives drugs (Pawlaczyk et al., 2011).

From the plant *Flaveria bidentis* (L.) Kuntze, the anticoagulant activity of its sulfated flavonoids, quercetin 3,7,3′,4′-tetrasulfate (QTS) and quercetin 3-acetyl-7,3′,4′-trisulfate (ATS) was investigated (Guglielmone et al., 2002). Thrombin time (TT), aPTT, antithrombin III (ATIII), PT, and heparin cofactor II (HCII) activation were measured. The flavonoids exhibited HCII activation by acting as agonists with higher activation observed exhibited by QTS than ATS (Guglielmone et al., 2002).

Marrubiin **(20)** was isolated from *Leonotis leonurus* (L.) R. Br., and both were tested *via in vivo* and *in vitro* studies to determine their anticoagulation reaction (Mnonopi et al., 2011). The marrubiin **(20)** and the plant extract suppressed the inflammatory markers, platelet aggregation, and coagulation marker while prolonged aPTT. The extract and marrubiin **(20)** in the concentration of 100 µg/mL were administered on rats. It was observed that the platelet adhesion was reduced in a dose-dependent manner, together with a depletion in protein secretion, fibrin formation, and d-dimer. The intracellular levels of Ca^2+^ were also reduced in a concentration-dependent manner and inhibited the calcium mobilization that was induced by thrombin by 50 to 200 µg/mL. This study shows that marrubiin **(20)** and the extract may have a direct inhibitory effect upon the synthase activity of cyclooxygenase or thromboxane due to the suppression of thromboxane B2 production (Mnonopi et al., 2011).

*Leuzea carthamoides* Willd. DC. consists of eriodictyol **(21)** and patuletin **(22)**, which have similar antiplatelet activity (Koleckar et al., 2008). The leaf parts of the plant exhibited antiplatelet activity by inhibiting arachidonic acid and collagen-induced platelet aggregation. It showed a more potent antiplatelet activity in the collagen-induced aggregation compared to the arachidonic acid-induced aggregation. The mechanism that was exhibited by eriodictyol **(21)** is the decrease in antiplatelet potency that was caused by glucosylation process. Based on the study, apigenin and quercetin formed from the glycosylation process exhibited a lesser activity compared to their aglycons (Guerrero et al., 2005). Based on the study, the extract of the *L. carthamoides* has a potent antithrombotic effect due to the presence of the antithrombotic agents, not due to the effect of the specific flavonoids that are present in the extract. The plant had shown a strong inhibition activity upon the platelet aggregation that was induced by adenosine diphosphate (ADP) (Koleckar et al., 2008).

*Salvia miltiorrhiza* Bunge consists of phenolic acid that is water-soluble, which is the salvianolic acid **(23)** (SAA). The study examined the effect of SAA in the antiplatelet and antithrombotic effect (Fan et al., 2010). In the *in vitro* assay, Tyrode’s solution was used to study the antiplatelet properties using a platelet aggregometer. The maximum height reached *via* the aggregation curves determines the extent of platelet aggregation of SAA. All tests showed that SAA had an inhibitory effect on ADP, thrombin, and platelet aggregation that was induced by AA. The compound inhibited ADP-induced platelet aggregation of rats with an IC_50_ value of 390 µg/mL, whereby it inhibited the thrombin-induced platelet aggregation with an IC_50_ value of 912 µg/mL. SAA (1,000 µg/mL) exhibited mild inhibitory activity on AA-induced platelet aggregation. In the *in vivo* study, the administration of SAA at dose 2.5, 5, and 10 mg/kg *via* intravenous caused dose-dependent inhibition upon the platelet aggregation in the rats. A similar observation with the *in vitro* study was observed. This study claimed that the SAA antiplatelet activity was due to the interference to a common signaling pathway, then directly binding to thrombin, ADP or AA to their respective receptors. The inhibition of ADP from dense granules of activated platelet might be one of the factors of the anti-aggregating properties of SAA (Fan et al., 2010).

The anticoagulant activity of *Tridax procumbens* (L.) L. was examined (Naqash and Nazeer, 2011). Sulfated polysaccharide isolated from the leaf extract of *T. procumbens* acts as an anticoagulant on heparin and chondroitin sulfate. Based on the *in vivo* assay, the activated aPTT had been prolonged to 113 s at 100 μg/mL by the sulfated polysaccharides from *T. procumbens*, which is almost 4-fold higher than the standard group. The sulfate group causes the anticoagulant activity but it is dependent on the sulfate group position in the chemical structure (Naqash and Nazeer, 2011).

### Diuresis Action

Diuresis is an important mode of treatment for cardiovascular diseases, such as hypertension. It can increase the urinary volume and has a fewer side effect compared to others. Diuretics usually used as an independent drug or a combination with other drugs of the same mechanism of action in easing various conditions such as congestive heart failure, ascites, and pulmonary edema as well. Thus, few of the available diuretics cause adverse effects such as an imbalance of electrolytes, alterations in metabolic status, and some may impair the sexual function (Gupta and Neyses, 2005; Morganti, 2005).

Based on the previous studies of Asteraceae and Lamiaceae plant species, the number of plant species that exhibits diuretic effects is lesser than those other mechanisms, such as antioxidants, antihyperlipidemic, and vasorelaxant. The plant species that possess diuretic effect from the Asteraceae family are *Chamaemelum nobile* (L.) All.*, Chrysanthemum* x *morifolium* Ramat. Hemsl., and *Tanacetum vulgare* L. and the plant species from Lamiaceae family are *Ajuga integrifolia* Buch.-Ham. ex D. Don (syn. *Ajuga remota* Benth.)*, Anisomeles indica* (L.) Kuntze, and *Plectranthus amboinicus* (Lour) Spreng. The diuretic effect of *C. nobile* was assessed by examining the rats’ urine after fasted overnight (Zeggwagh et al., 2009). After repeated oral administration of 140 mg/kg aqueous plant extract for three weeks, the extract showed a diuretic and hypotensive effect.

*Plectranthus amboinicus* (Lour) Spreng is a plant species from the Lamiaceae family. Its leaf aqueous, alcoholic, and ethyl acetate extracts increased urine volume and decreased serum sodium level of albino rats after 24 h compared to the moduretic drug (El-Hawary et al., 2012). Meanwhile, the extracts did not show any significant effect on the rats’ potassium level. Based on the study, the ethyl acetate fraction was more potent as a diuretic group with better electrolyte balance (El-Hawary et al., 2012).

*Tanacetum vulgare* L. leaf extract was studied on its ability to act as a diuretic on rats (Lahlou et al., 2007). Water extract of the plant at a dose of 100 mg/kg was administered orally to the male Wistar rats. An increase in urine output was identified after 24 h of administration of the extract with a similar amount compared to furosemide administration. The extract had caused an increased level of Na^+^ and K^+^ in the urine, compared to furosemide, which has only an increase in Na^+^. In contrast, the extract does not affect Na^+^ and K^+^ levels of the plasma. The diuretic effect occurs due to the renal tubular suppression upon its tendency for reabsorption of electrolytes and water into the bloodstream. The plant extract does not cause any renal toxicity as repeated dosing upon the rat for nine consecutive days. Thus, it requires a clinical study to ensure that its safety profile matches up with the physiology of humans and the long duration that patients usually take on diuretics. Different studies have shown that *T. vulgare* consists of flavonoids, tri- and sesquiterpene lactones and isoprenoids, polysaccharides, saponins, and polyphenols. Thus, it is unsure of which compound contributes to the diuretic effect of this extract (Lahlou et al., 2007).

## Toxicological Studies

There are plenty of pharmacological studies on the Asteraceae and Lamiaceae plant species on its cardiovascular effects, while the toxicological aspects of these species have yet to be explored. Most of the people believe that medicines that are from medicinal plants or herbal medicines are always safe, simply due to the belief that all plants are safe to be consumed. This does not apply to all the medicinal plants. Medicinal plants have their toxicity nature depending on their dosage and method of extraction (Chanda et al., 2015).

Based on the studies in this review, few had included its toxicology test results and had herbal drug interaction during the administration of the medicinal plant extract in specific conditions. Traditional preparation methods of the medicinal plants into a consumable substance take different toxicity levels. The level of toxicity differs according to the solvents used. Thus it is important to choose the appropriate solvent either for *in vivo* or *in vitro* experiments.

Acute toxicity test of *Artemisia campestris* L. showed no symptoms of toxicity upon its aqueous extract administration of doses 1, 2, 4, and 6 g/kg (Dib et al., 2017).Based on the acute toxicological studies on *C. crepidioides* (Benth.) S. Moore, it showed that consumption of the plant extract at a dose of 2500 mg/kg was safe. During the observation on the first 8 h, within the interval of every 8 h and upon the next 72 h, no significant change in the animal behavior or mortality was observed (Bahar et al., 2016). It showed that the dose is safe in the *in vivo* testing.

Acute toxicity study was conducted on the ethanolic extract of *Launaea intybacea* (Jacq.) Beauverd (syn. *Lactuca runcinata* DC.) in 1% gum acacia upon rats (Devi and Muthu, 2015). Subsequent administration of the extract at dose 2,000 mg/kg body weight of rats for 14 days showed no toxicity effect, and this dose had helped in the reduction of total cholesterol and elevated the HDL level.

The plant in this study that showed toxicity is *Leonurus cardiaca* L. Lavandulifolioside, the active component of *L. cardiaca*, showed moderate toxicity at the amount of 1,000 mg/kg on the LD_50_ when given intravenously (Wojtyniak et al., 2013). The butanol extract of the plant showed higher toxicity with LD_50_ of 400 mg/kg when administered intravenously compared to LD_50_ of 2,000 mg/kg when administered orally. This showed that the intake of this drug *via* intravenous possess a higher toxicity possibility than *via* oral intake (Wojtyniak et al., 2013).

Aqueous extract of *Salvia scutellarioides* Kunth was administered on mice in two doses of 1 or 2 g/kg for 28 days (Ramirez et al., 2007). The administration showed no mortality. The study also claimed that if *S. scutellarioides* is taken together with other diuretics drug, it might worsen hypokalemia symptoms or increase in digoxin related arrhythmias in patients because of the herb-drug interactions. (Ramirez et al., 2007). *Teucrium polium* L. is known for its risk of hepatotoxicity due to hepatocyte necrosis occurs massively in the central lobular area such as lymphocyte inflammatory inflate, bile duct proliferation, and bile retention. Clinical signs are often seen in the usage of this plant; thus, proper usage of this plant on the therapeutic range would resolve the problem.

## Conclusion

The last few decades have witnessed several rapid changes in the traditional use of the medicinal plant in developing countries. However, some of the traditional use of the medicinal plant is undocumented that results in the decline of knowledge and making it unreliable. Therefore, it has become necessary to document the knowledge and shared them entirely to ensure their quality and preservation. Based on this review, medicinal plants were widely consumed using decoction or taken orally as a raw product of fruits, leaves, and roots. Most of the plants from Asteraceae and Lamiaceae family are rich in flavonoids and terpenoids, together with other phytochemicals that act as the inducer of the mechanism in alleviating cardiovascular diseases. The plants have a strong antioxidant effect, followed by anti-hyperlipidemic, vasodilation, antithrombotic, and diuretic effects which are mechanisms that are closely related in resolving cardiovascular diseases such as coronary heart diseases (CHD), atherosclerosis, hypertension, and others. As the medicinal plant being beneficial for treating human ailments, we should not waste these resources by leaving them to grow wild and perish, without utilizing them for better pharmaceutical development in the future.

## Future Studies

Based on the evidence-based review on the use of medicinal plant from the plant family of Asteraceae and Lamiaceae in cardiovascular diseases, we hope that information from this review will facilitate future research initiatives to develop new medicinal plant-based medication for cardiovascular disease treatment or continue with any clinical studies to prove the effectiveness of this medicinal plant upon humans. The clinical trial needs to be performed to have a better knowledge of their safety and efficacy to ensure that it can be beneficial to the human race.

## Author Contributions

JM obtained the pieces of literature and wrote the manuscript while NA and KH edited the manuscript.

## Conflict of Interest

The authors declare that the research was conducted in the absence of any commercial or financial relationships that could be construed as a potential conflict of interest.
